# Supplementary material for: Signal improved ultra-fast light-sheet microscope for large tissue imaging
Source: Commun Eng. 2024 Apr 2;3:59. doi: 10.1038/s44172-024-00205-4 (PMC10987599; doi:10.1038/s44172-024-00205-4)
Supplement: Supplementary file 1 — Supplementary information [file 44172_2024_205_MOESM1_ESM.pdf]

Signal Improved ultra-Fast Light-sheet Microscope for large tissue imaging

Md Nasful Huda Prince[1], Benjamin Garcia[2], Cory Henn[2], Yating Yi[3], Etsuo A. Susaki[4], Yuki Watakabe[5,6], Tomomi Nemoto[5,6], Keith A Lidke[1], Hu Zhao[3], Irene Salinas Remiro[2], Sheng Liu[1], Tonmoy Chakraborty[1,7]*

[1]Department of Physics and Astronomy, University of New Mexico, Albuquerque, NM 87131, USA
[2]Department of Biology, University of New Mexico, Albuquerque, NM 87131, USA
[3]Chinese Institute for Brain Research, Bejing 102206, China
[4]Department of Biochemistry and Systems Biomedicine, Graduate School of Medicine, Juntendo University, Tokyo, Japan
[5]Division of Biophotonics, National Institute for Physiological Sciences, National Institutes of Natural Sciences, 5-1 Higashiyama, Okazaki, Aichi, 444-8787, Japan
[6]Biophotonics Research Group, Exploratory Research Center for Life and Living Systems, National Institutes of Natural Sciences, 5-1 Higashiyama, Okazaki, Aichi, 444-8787, Japan
[7]Comprehensive Cancer Center, University of New Mexico, Albuquerque, NM 87102, USA

*Corresponding authors, tchakraborty@unm.edu

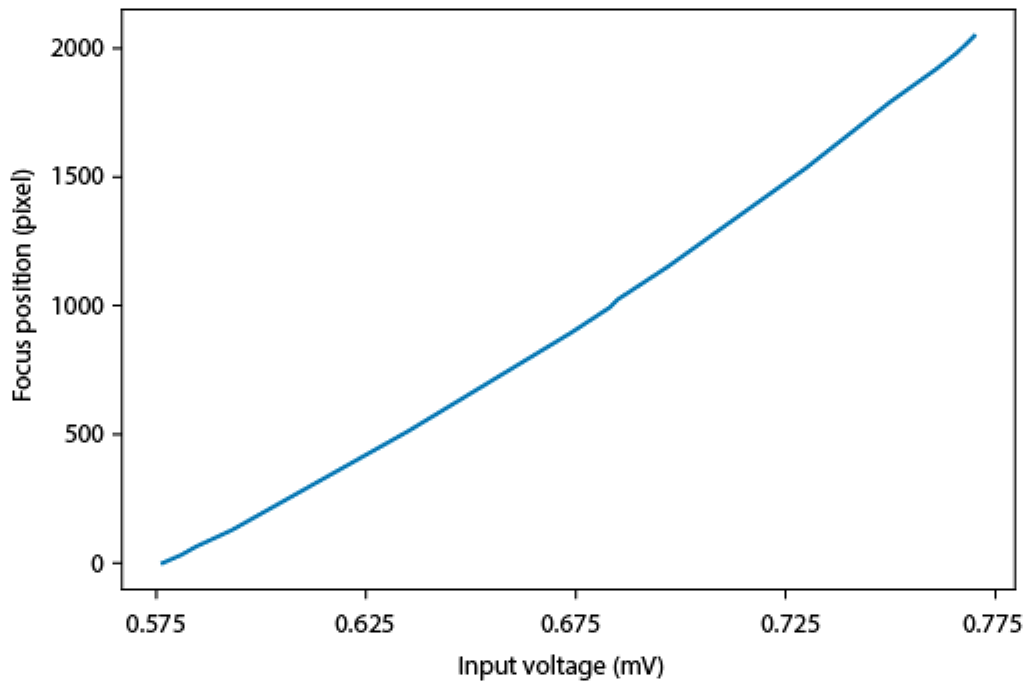

Supplementary Figure 1 | **LFA operating region.** Linear LFA movement with respect to the input voltage.

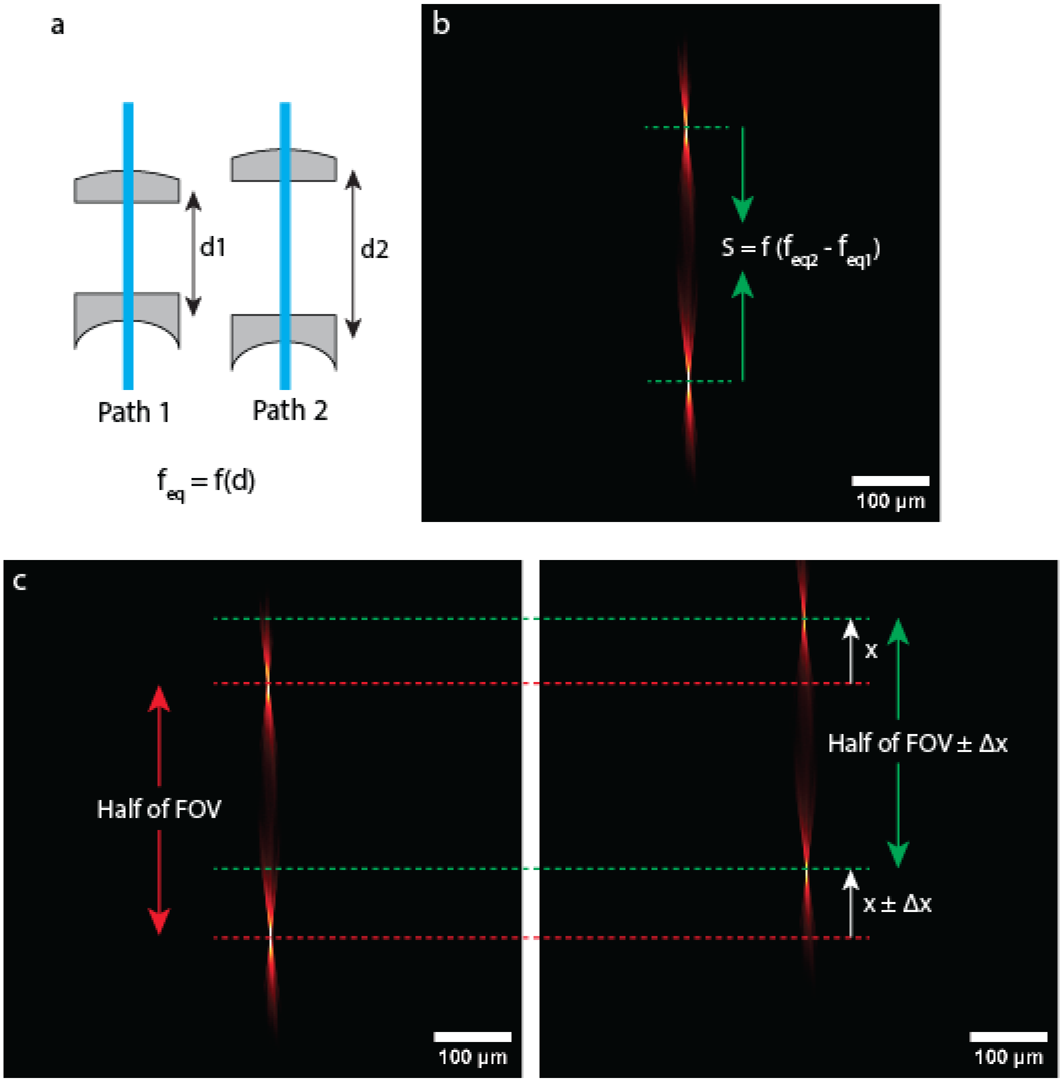

Supplementary Figure 2 | Challenges in previous work that are alleviated by SIFT. a-b, Lens pair combinations (**a**) caused different effective focal lengths (**b**) which is responsible for creating the separation between the two foci. **c,** Differential magnification of the two light paths prohibited us from keeping the fixed separation between the two foci while moving throughout the FOV. For example, if the initial foci (red dashed lines) are separated by “half of the FOV”, the differential magnification causes a separation of “half of the FOV ± Δx” at the next instance (green dashed lines) which eventually destroys the synchronization between the foci and the camera rolling shutter.

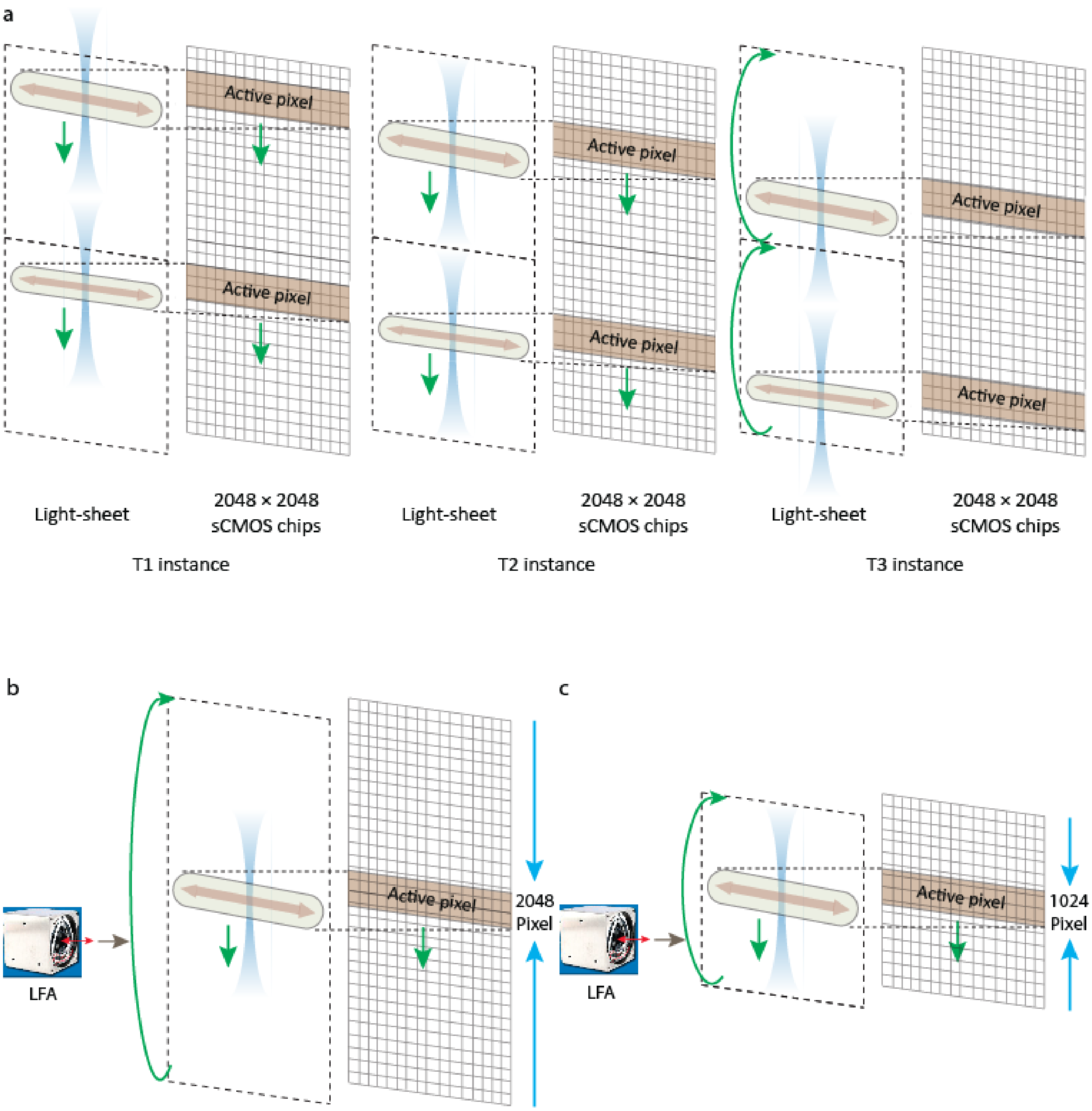

Supplementary Figure 3 | 2D focus scanning for axial sweeping. a, Synchronous movement of dual 2D focus with the sCMOS camera rolling shutter, keeping a fixed separation between the two foci for three different instances. The beam waists are captured by the camera chip. **b-c,** The mechanical movement of LFA shaft causes the focus to move in the sample space. The range of the shaft's movement for single focus is twice (**b**) compared to the dual foci arrangement (**c**).

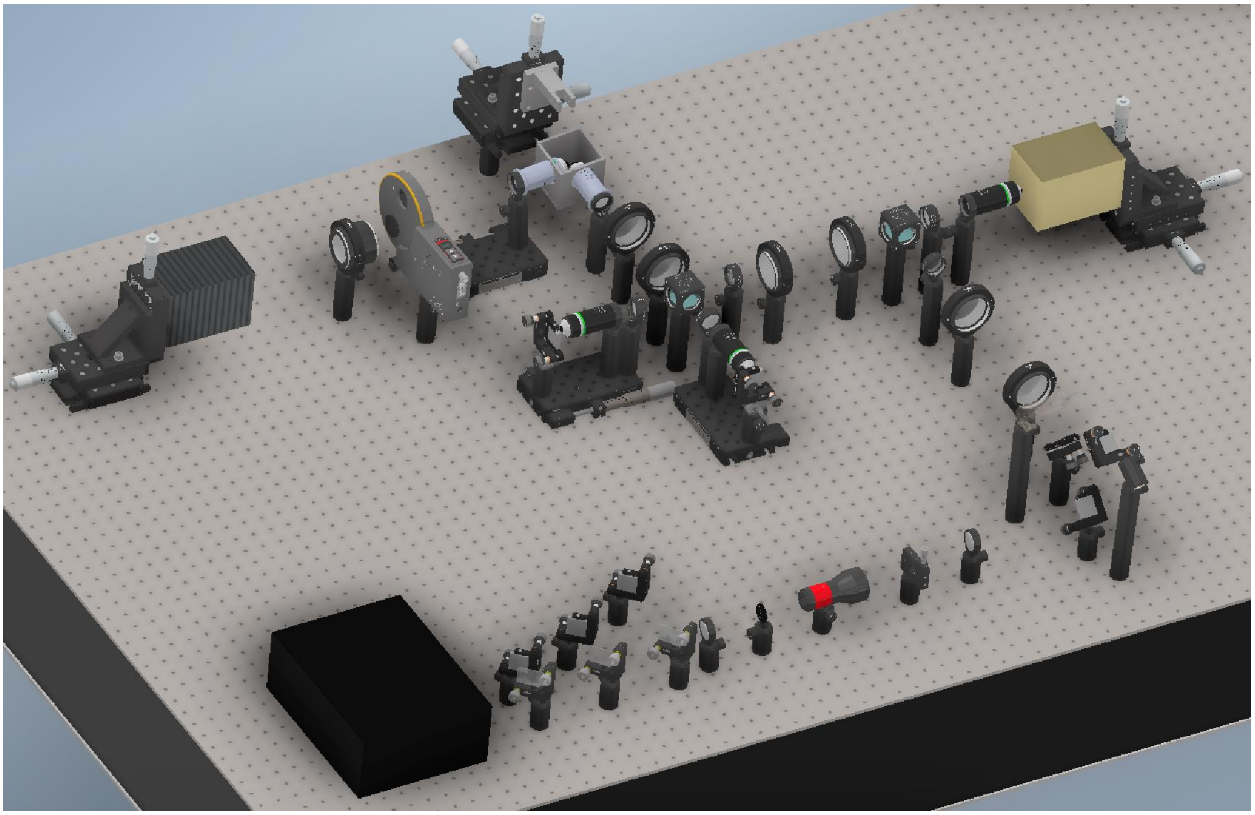

Supplementary Figure 4 | **Experimental implementation of SIFT.** 3D experimental implementation of SIFT. The necessary equipment list of SIFT implementation is delineated in **Supplementary Table 1**.

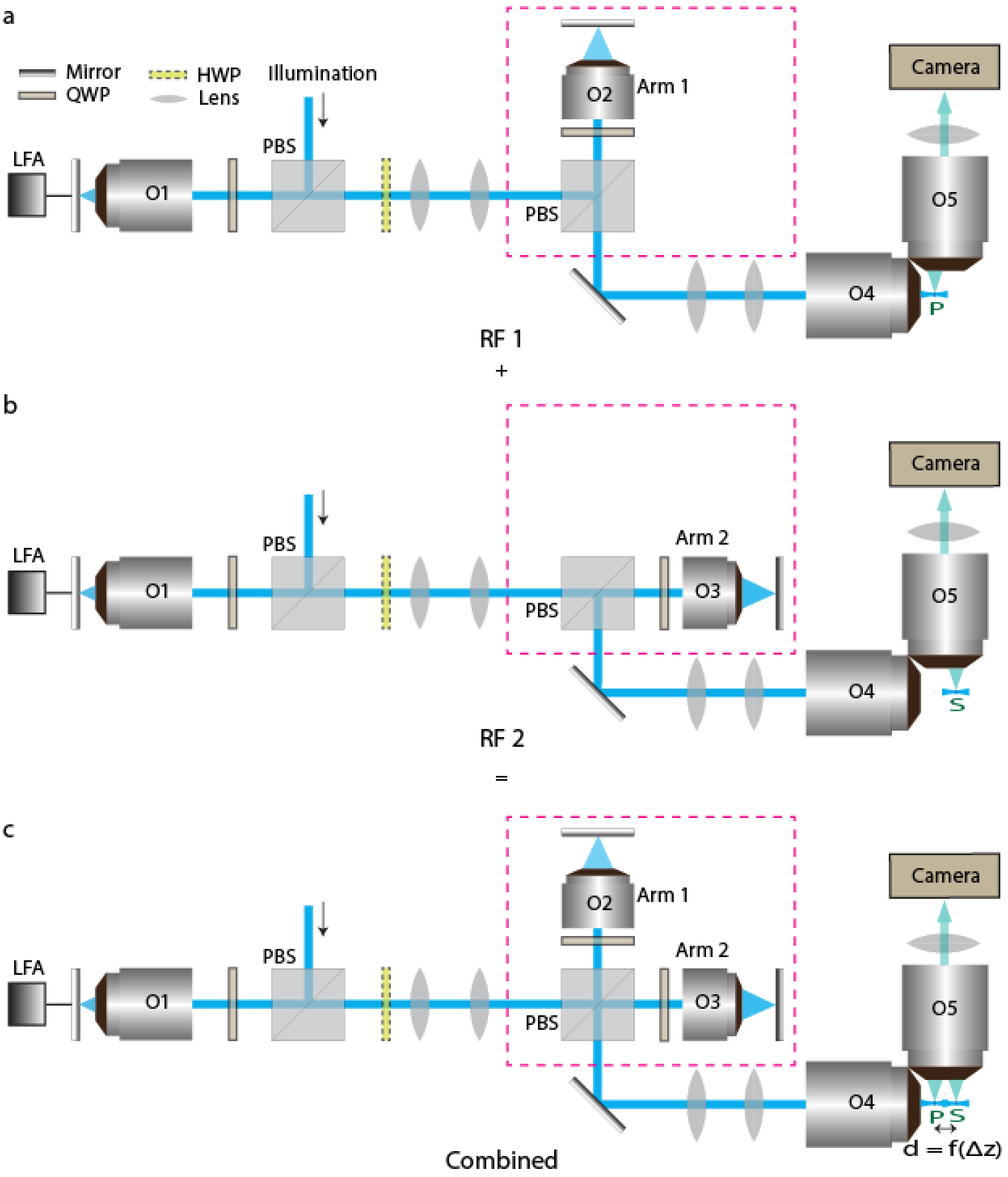

Supplementary Figure 5 | **Generation of two staggered light-sheet. a-b,** Two identical remote focusing (RF) path (RF1 and RF2). The RF1 includes O1, O2 and O4 where O1 and O2, O2 and O4 are pupil matched using the lens pair between them (**a**). Again, RF2 includes O1, O3 and O4 where O1 and O3, O3 and O4 are pupil matched (**b**). **c,** Combining RF1 and RF2 generates two LSs in the sample space. Different defocus parameter of the two path introduces a fixed distance between the two foci.

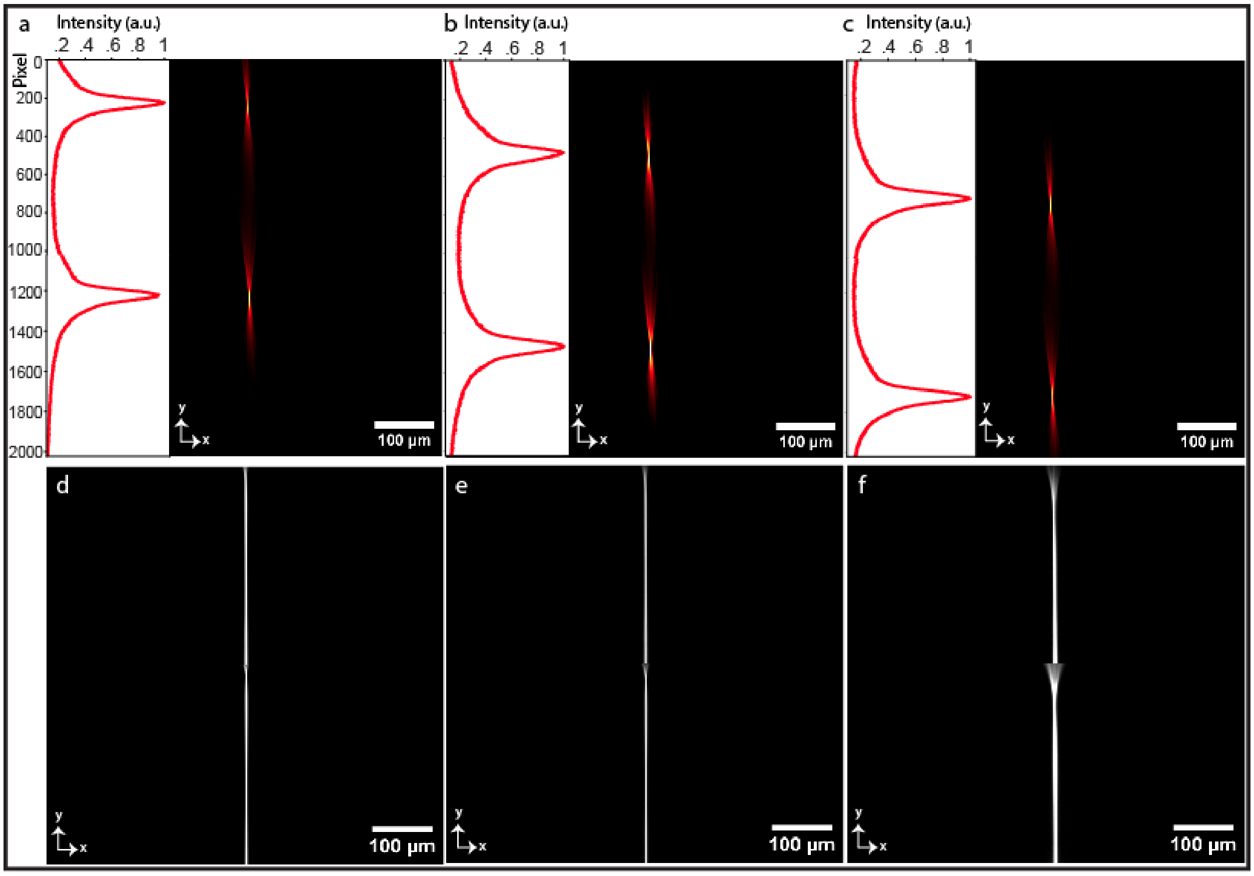

Supplementary Figure 6 | Foci synchronization with camera rolling shutter. a-c, 2D foci along with the vertical intensity profile for the various positions of the camera FOV. The intensity profile ensures no out-of-focus light overlaps between the two LSs while travelling throughout the FOV. **d-f,** Scanning of 2D focus across the entire FOV for 25 ms (**d**), 20 ms (**e**) and 10 ms (**f**) of camera exposure time. The sharps line across the entire FOV depicts the tight synchronization between LSs and the rolling shutter which ensures uniform resolution across the entire FOV. It is also proven from **e** and **f** that with a small compromise of the FOV we may achieve the uniform resolution for even 20 ms or 10 ms of camera exposure time, five or ten-fold shorter than the exposure time of traditional ASLM.

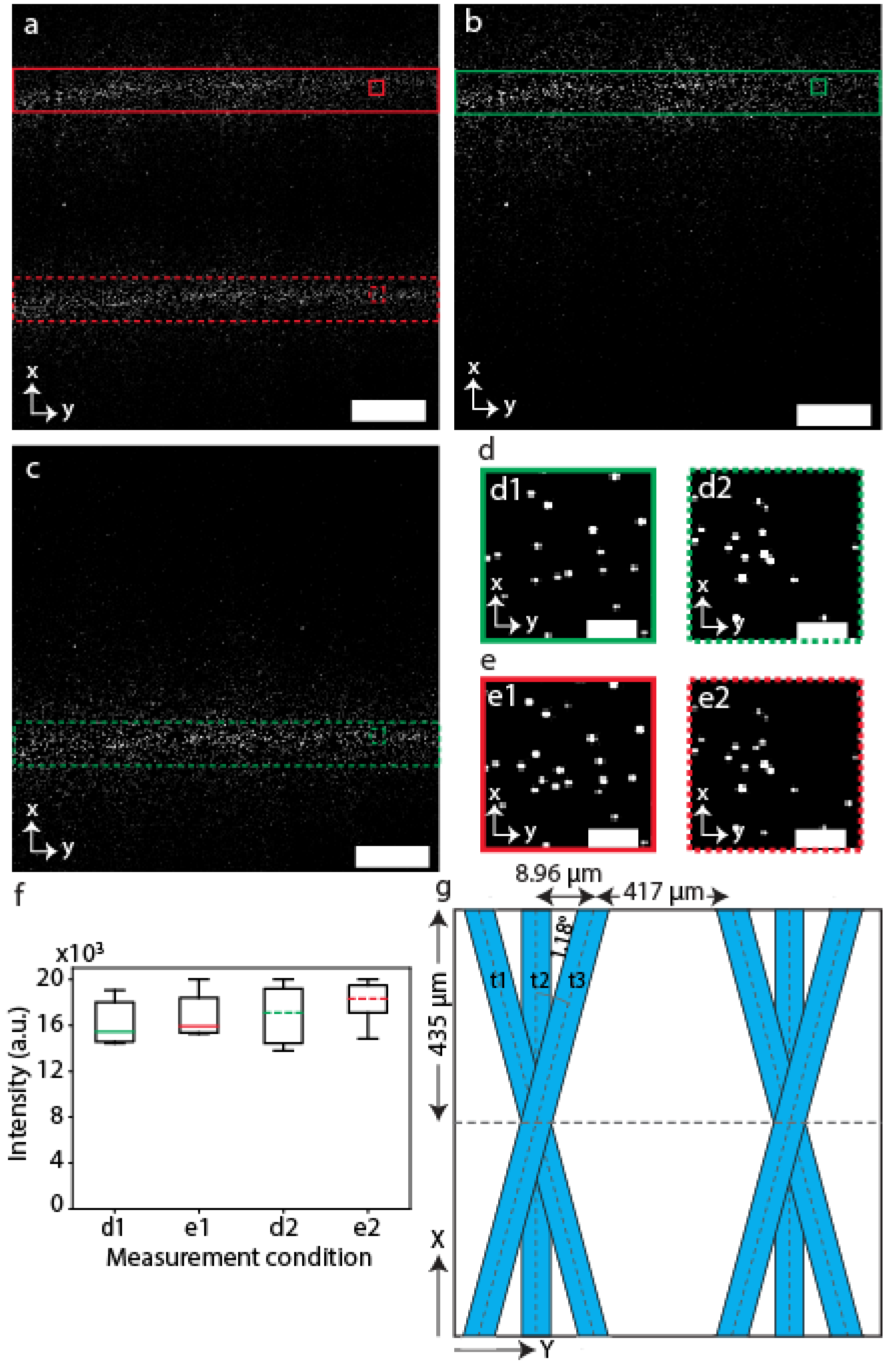

Supplementary Figure 7 | Defocus bleed through measurement using 500 nm fluorescent beads. a-c, Maximum intensity projection (MIP) of 500 nm fluorescent beads embedded in 2% agarose and submerged in water while both LSs are active (**a**), only the top LS is active and the bottom one is blocked (**b**), only bottom LS is active and the top one is blocked (**c**). The experiment was conducted for static Gaussian LS (not in ASLM mode i.e. no LS movement in Y) while the resonant galvo was dithering the LS in X direction. The red and green rectangle region delineates the waist of the corresponding LS. **d-e,** Zoomed-in view of a randomly selected region from around the edge of the X dimension of **b** and **c** (**d**), and **a** (**e**) where the bead from top and bottom provides approximately similar intensities. Beads from the top LS region of **a** and **b** are shown in the corresponding red and green square boxes (**e1** and **d1**) respectively, and the bottom LS region of **a** and **c** are shown in the corresponding red and green dotted square boxes (**e2** and **d2**). **f,** The maximum intensity of the beads taken from the region, shown in **d** and **e**, for each condition (**d1**, **d2**, **e1** and **e2**) delineates negligible bleed-through from one LS to another (n = 10). **g,** The LS was pivoted using a resonant galvo in X direction at 4 kHz frequency over a small angle (approximately 2.36°). This may cause the LS waist to broaden by about 9 µm leaving about 417 µm intermediate distance between the two LSs. Scale bars, 150 µm (**a,b,c**), 8 µm (**d,e**).

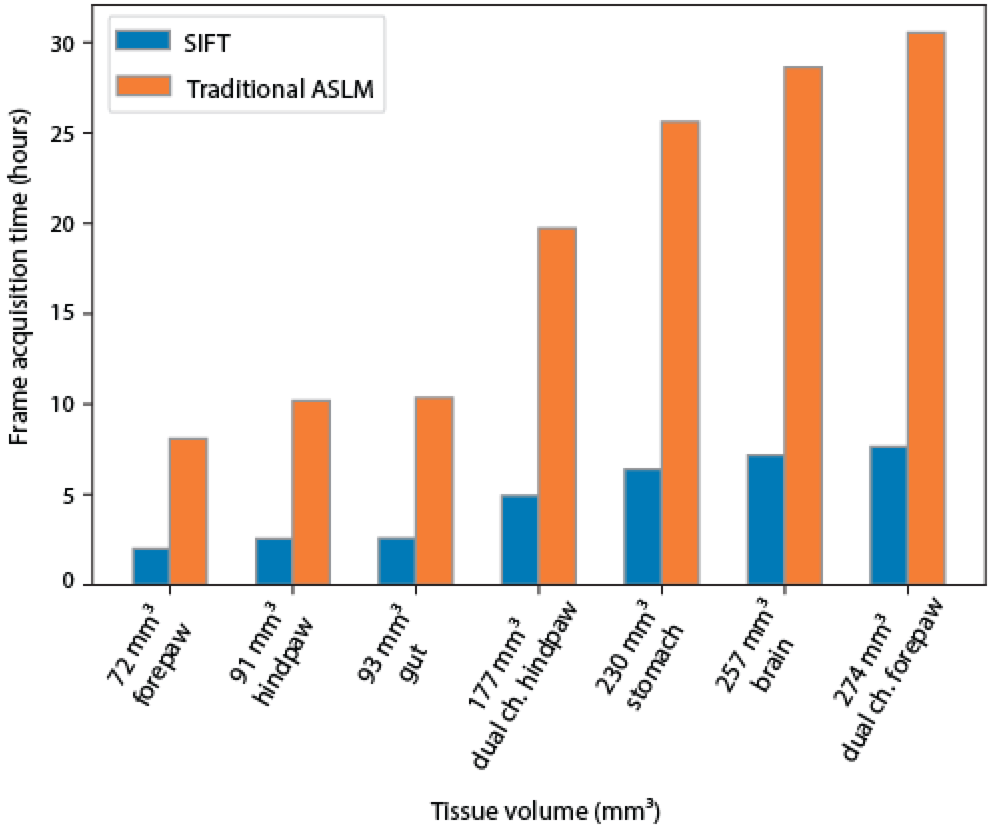

Supplementary Figure 8 | Frame acquisition time. Comparison of frame acquisition time for various tissue specimens having different shapes and volumes. The plot only considers the high-resolution frame acquisition time for both SIFT and traditional ASLM system. SIFT offers four fold improvement of frame acquisition time compared to the traditional system.

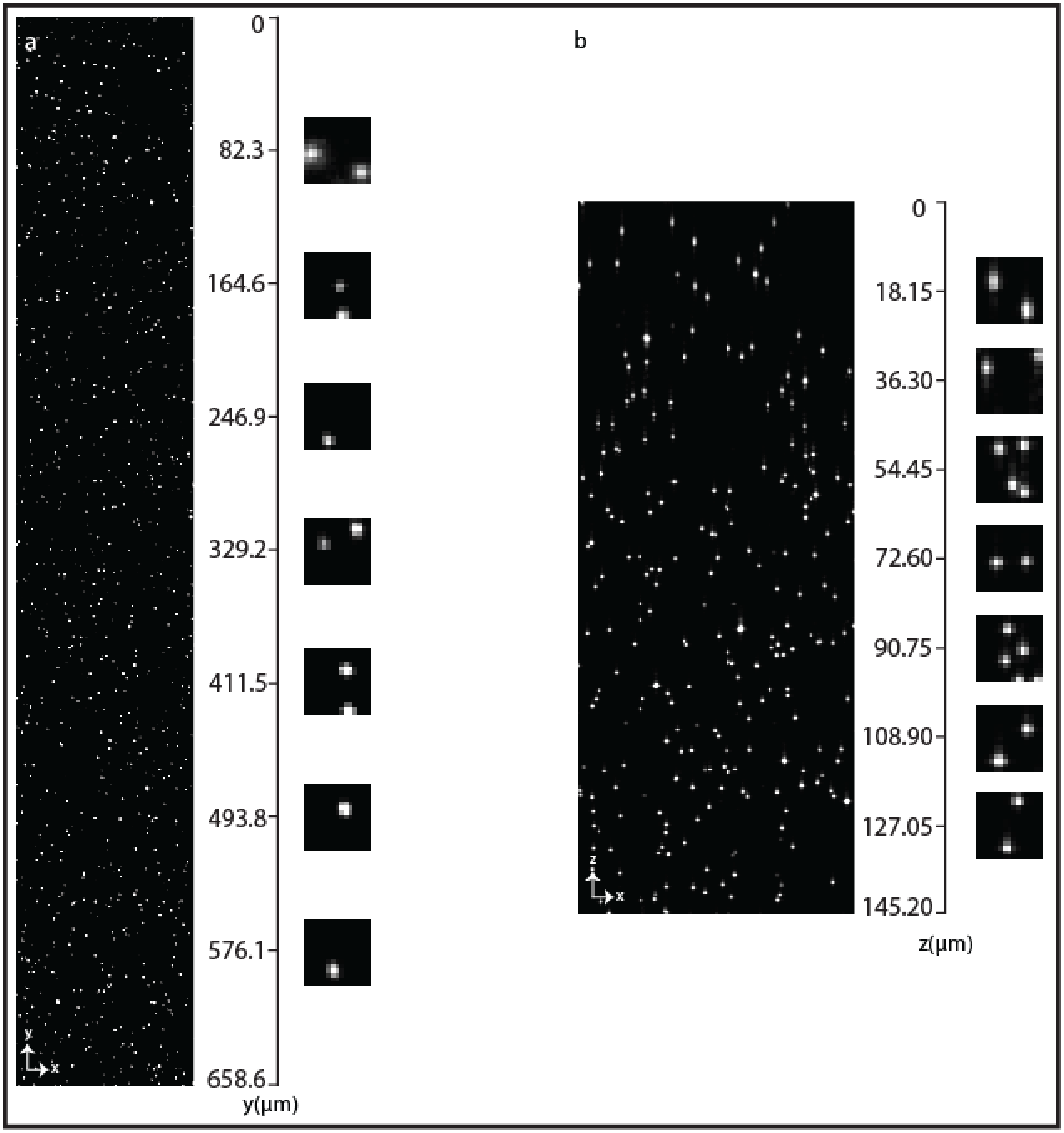

Supplementary Figure 9 | PSF uniformity. a-b, 500 nm fluorescent beads in lateral (**a**) and axial (**b**) direction. The beads were embedded in 2% agarose and submerged in water for imaging. The lateral distribution delineates the coverage of the entire FOV and the axial distribution illustrates the coverage over 145 μm of axial range.

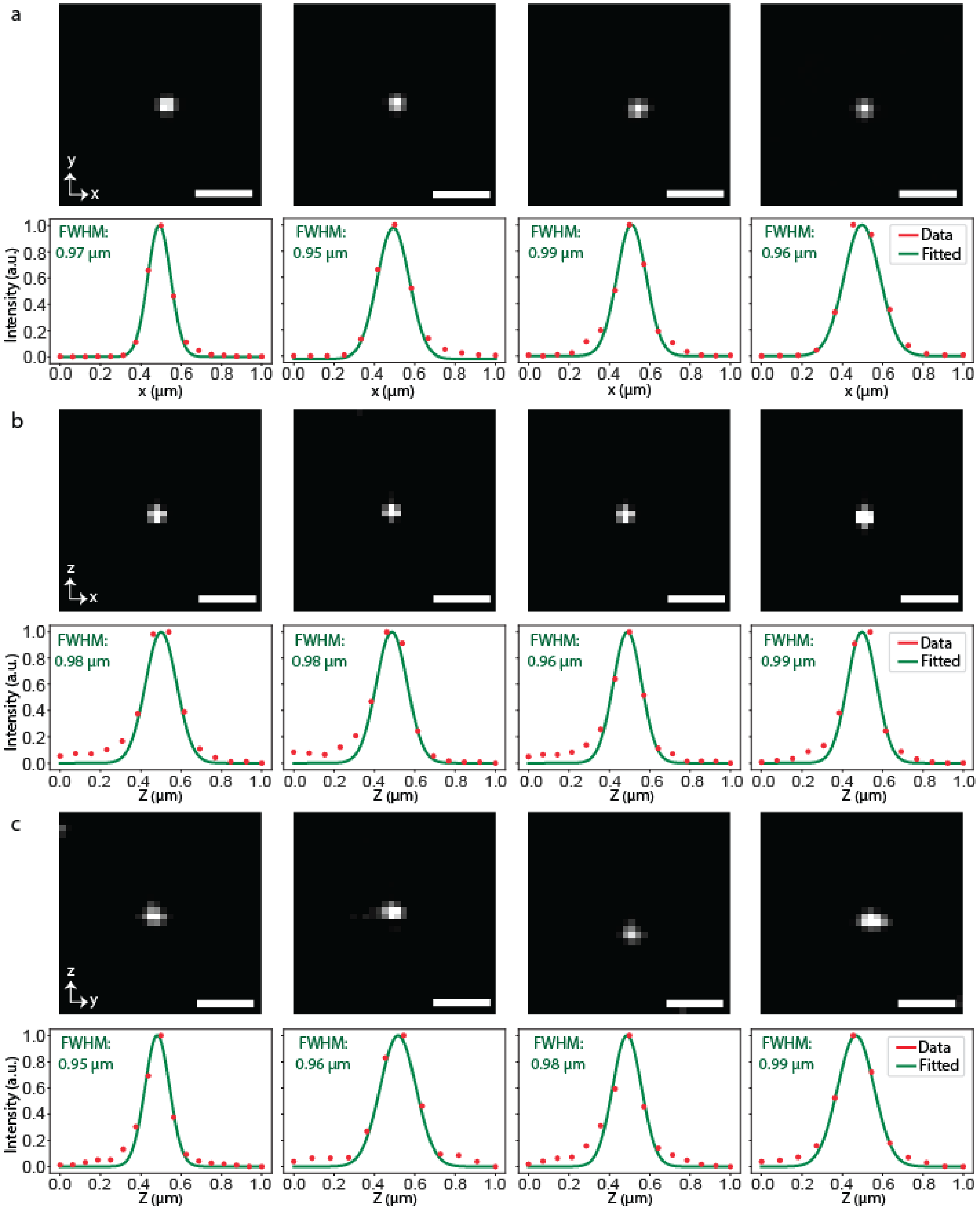

Supplementary Figure 10 | Isotropic PSF. a-c, SIFT enables imaging with isotropic resolution. 1st row shows four 500 nm fluorescent beads (imaged in water) and the 2nd row shows the FWHM of the PSF in XY (**a**), XZ (**b**) and YZ (**c**) dimensions. The isotropic imaging modality of SIFT provides the resolution of around 0.97 ± 0.05 µm in all dimensions. Scale bars, 3 µm (**a-c**).

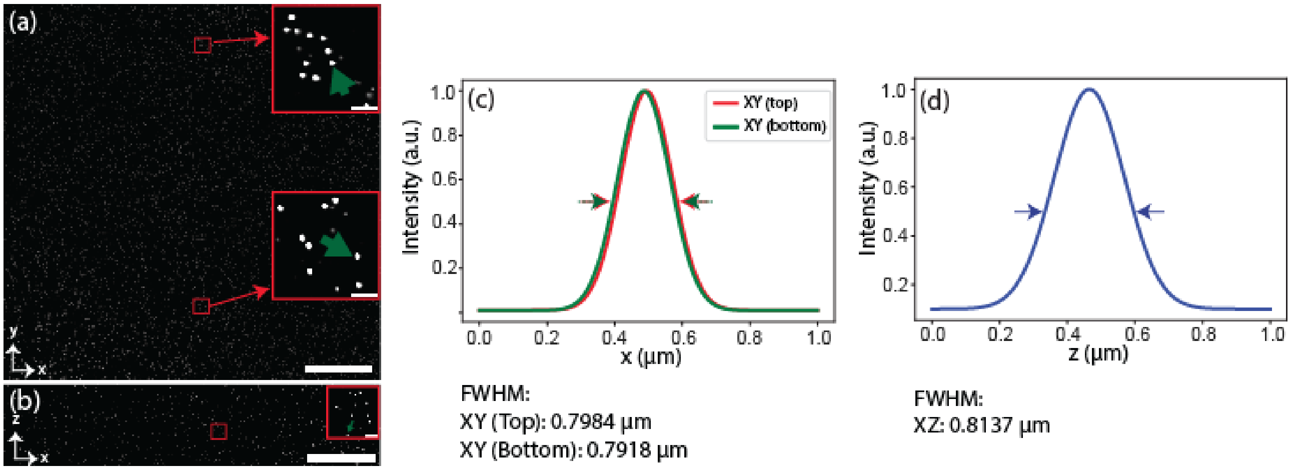

Supplementary Figure 11 | Deconvolved PSF. a-b, Distribution of 500 nm deconvolved (Richardson-Lucy) fluorescent beads image over the entire FOV both in lateral (**a**) and axial (**b**) dimension. The inset depicts the higher magnification view of a bead taken from a randomly selected area, shown in red colored rectangle. **c-d,** Profile plot of a randomly selected beads for lateral (**c**) and axial (**d**). The lateral beads are taken randomly from top and bottom half of the FOV and marked by green arrow-heads as shown in the inset of (**a**). The axial bead is also taken in a random basis as shown in green arrow-head of inset of (**b**). The corresponding FWHM (~ 0.80 ± 0.06 μm (n = 75)) proves that the deconvolution sharpened the images both in lateral and axial dimension.

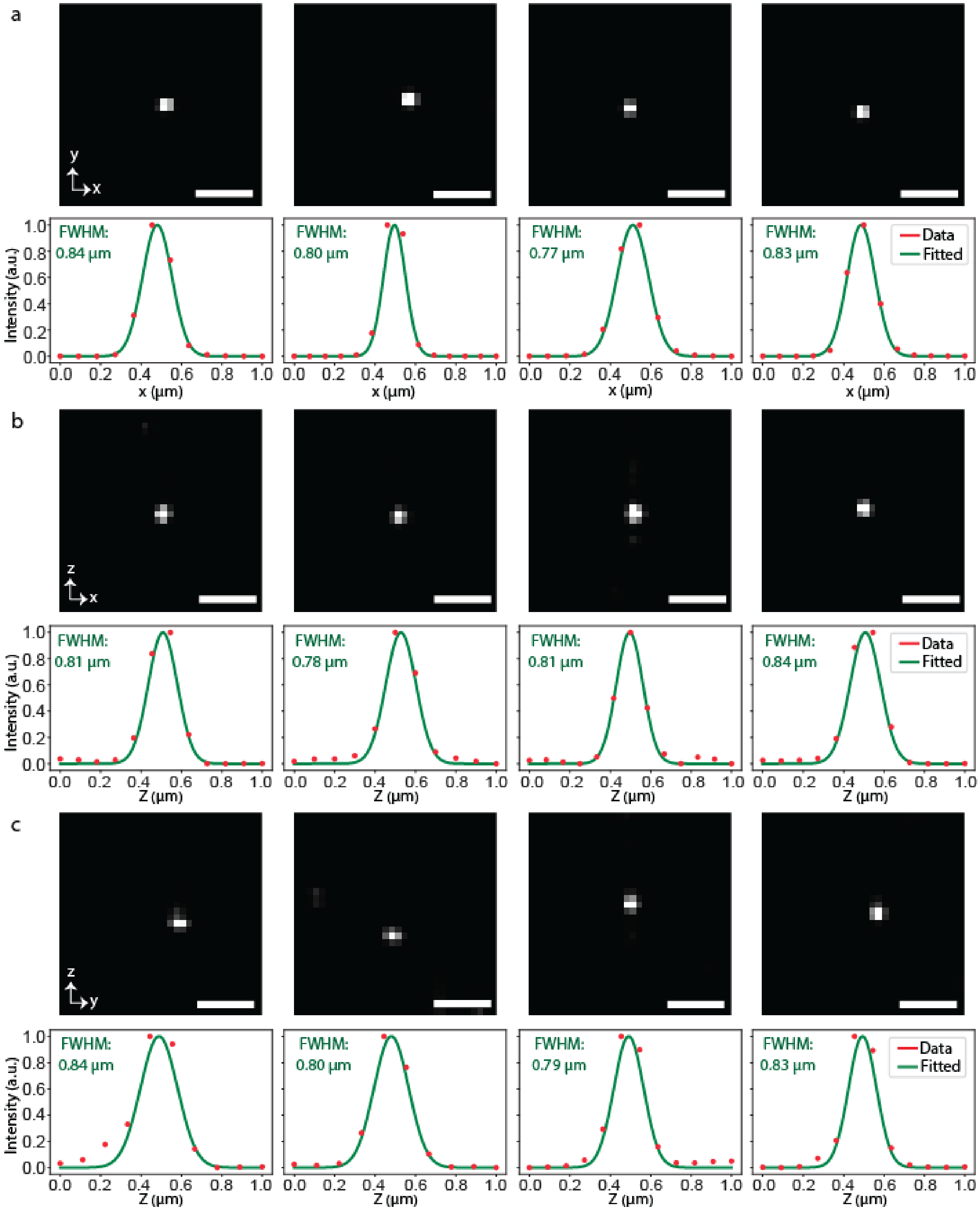

Supplementary Figure 12 | **Deconvolved isotropic PSF. a-c,** FWHM improvement following deconvolution. Several deconvolved (Richardson-Lucy) 500 nm fluorescent beads (imaged in water) are shown in the 1st row and the 2nd row shows the FWHM of the deconvolved PSF in XY (**a**), XZ (**b**) and YZ (**c**) dimensions. The isotropic imaging modality of SIFT provides the deconvolved FWHM of around 0.80 ± 0.06 µm in all dimensions. Scale bars, 3 µm (**a-c**).

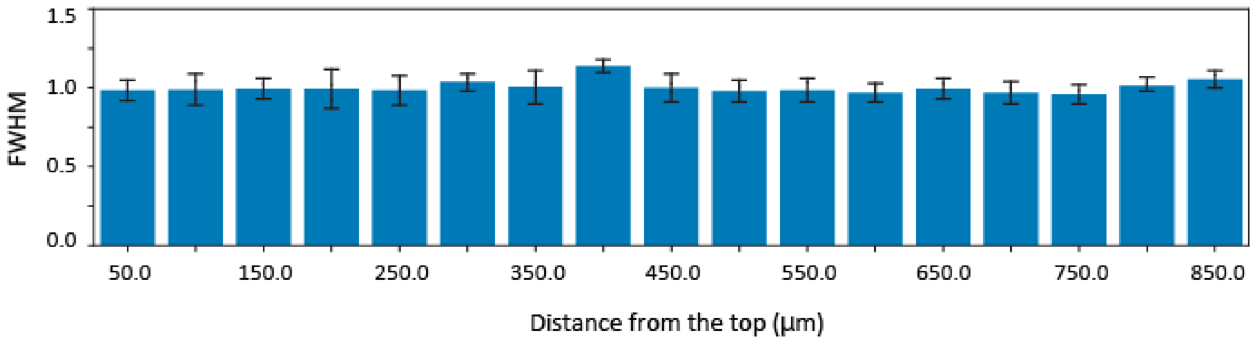

Supplementary Figure 13 | FWHM of 500 nm fluorescence beads as a function of the vertical distance over the FOV for SIFT at 25 ms of camera frame-rate.

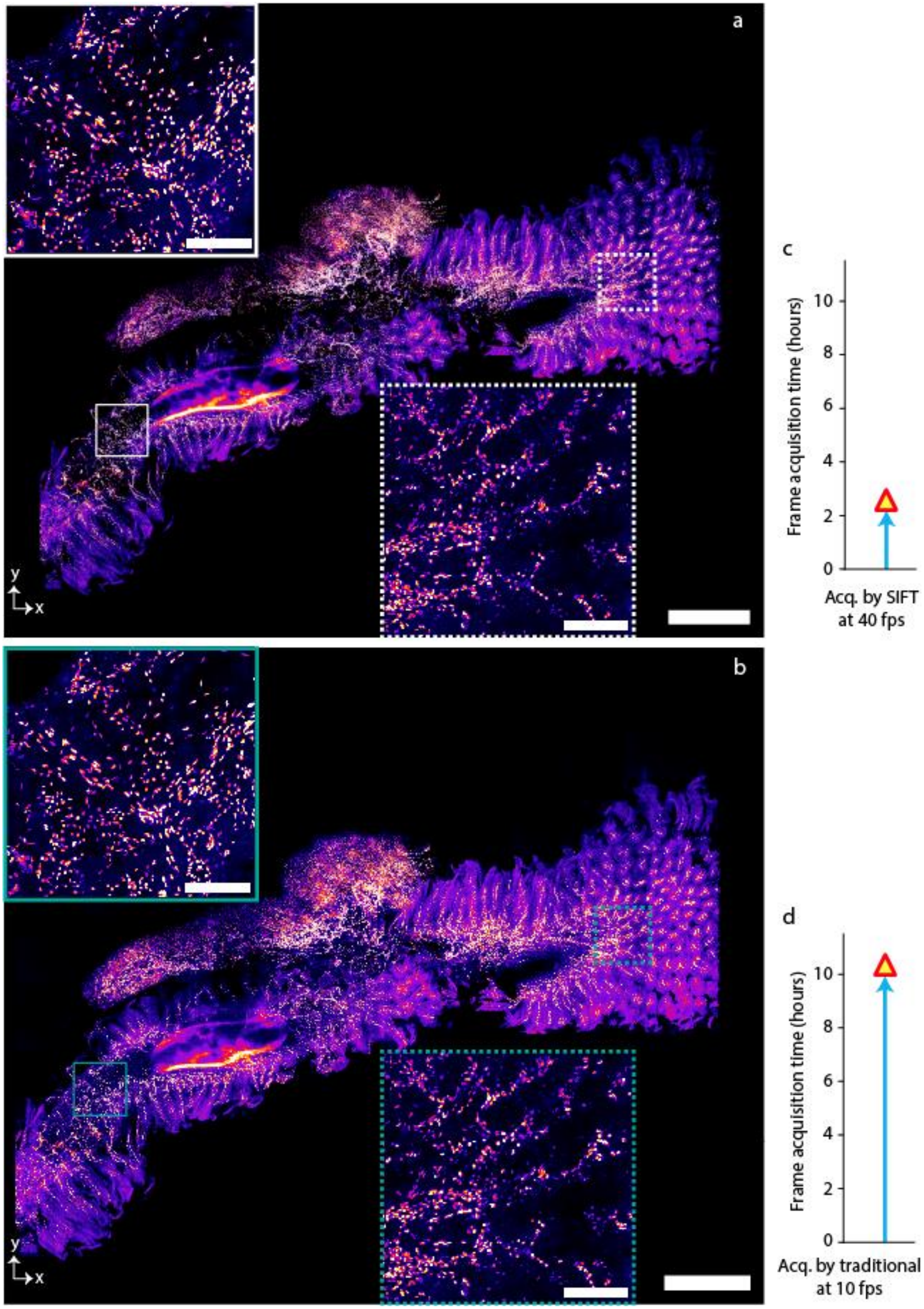
a
b
c
d
y
x
Frame acquisition time (hours)
0
2
4
6
8
10
Acq. by SIFT at 40 fps
Acq. by traditional at 10 fps

Supplementary Figure 14 | Volumetric imaging of mouse gut. a-b, MIP of 50 slices of Cdh5-cre Ai14 H2GFP mouse gut cleared by PEGASOS protocol and imaged by SIFT at 40 fps (**a**) and by traditional ASLM at 10 fps (**b**), inset shows the enlarged view of 2 randomly selected regions of (**a**) and (**b**). The image volume is 5.5 x 4.6 x 2.8 mm^3. Number of high resolution tiles that were required to image this volume was 878 tiles which generates 2.84 TB of image data. **c-d,** Comparison of total frame acquisition time to image the tissue between SIFT at 40 fps (**c**) and traditional ASLM at 10 fps (**d**). Scale bars, 600 µm (**a**), 100 µm (inset of **a**).

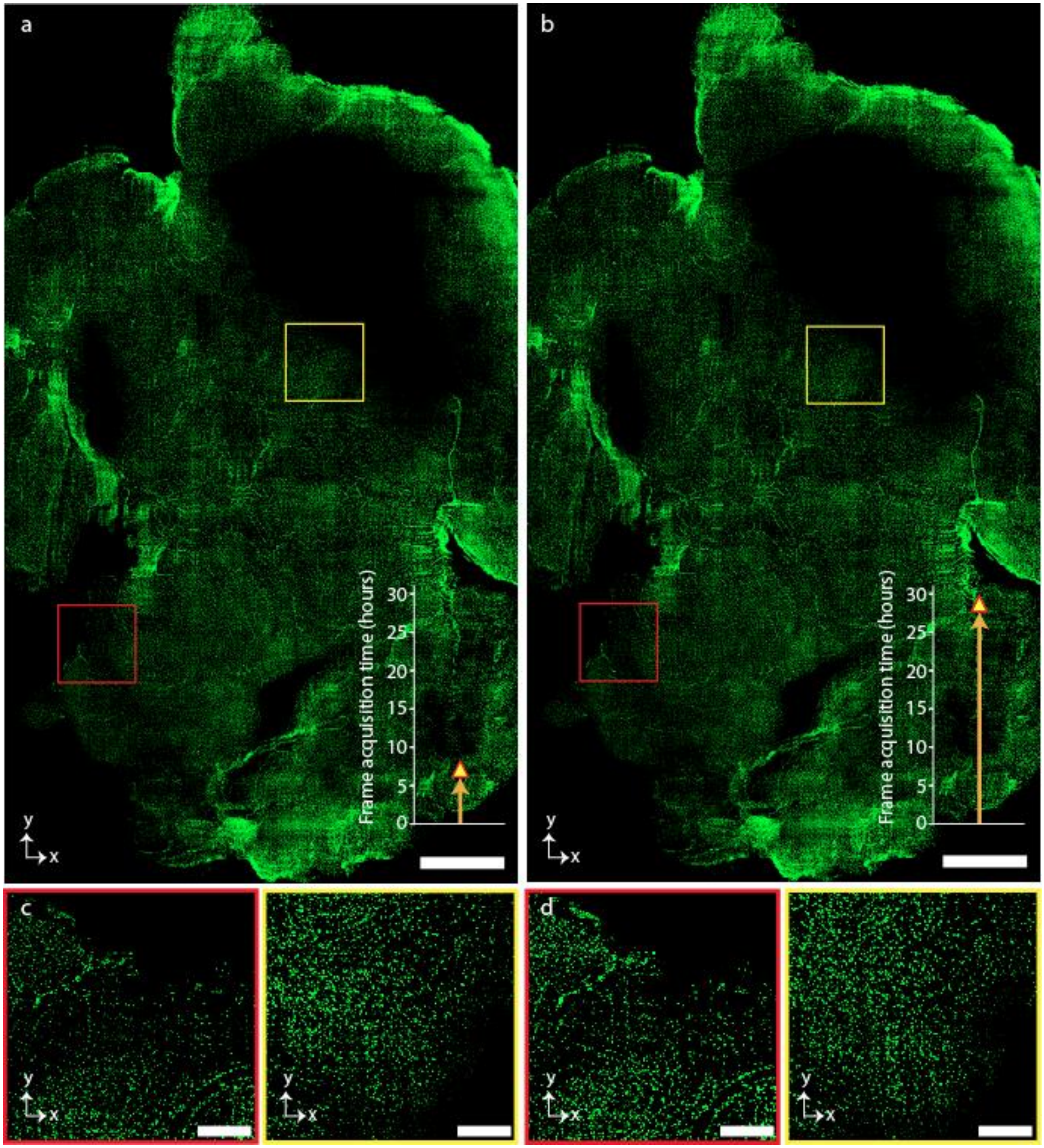

Supplementary Figure 15 | Volumetric imaging of mouse brain. a-b, MIP of 86 slices of Cdh5-cre Ai14 H2GFP mouse gut cleared by PEGASOS protocol and imaged by SIFT at 40 fps (**a**) and by traditional ASLM at 10 fps (**b**). The image volume is 10.4 x 6.1 x 3.2 mm^3. Number of high resolution tiles that were required to image this volume was 2,426 which generates 7.86 TB of image data. The bar plot attached to each image depicts the total frame acquisition time for the corresponding imaging platform. **c-d,** Higher magnification view of the randomly selected regions of **a** (**c**) and **b** (**d**) marked by corresponding red and yellow square boxes. Comparison of image acquisition time between SIFT at 40 fps (**c**) and traditional ASLM at 10 fps (**d**). Scale bars, 1 mm (**a**), 200 µm (**a,b**).

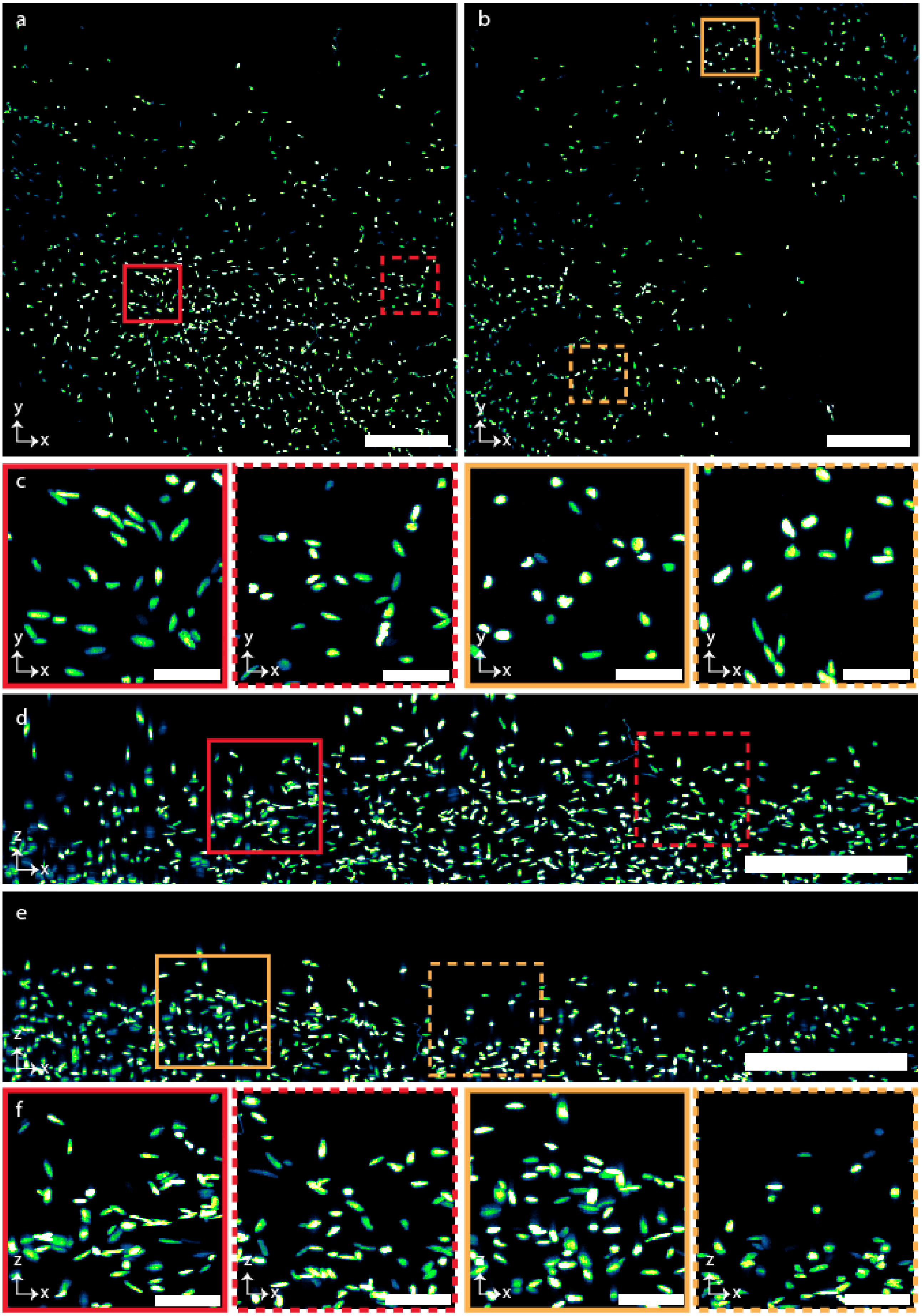

a
b
c
d
e
f
y
x
z

Supplementary Figure 16 | Imaging of nuclear stained mouse brain. a-b, Lateral view of 2 randomly selected mouse brain (**Supplementary Figure 15**) tiles imaged by SIFT at 40 fps. **c,** Higher magnification view of randomly selected regions from the 2 tiles shown in **a** and **b**, marked by the corresponding colored square boxes. **d-e,** Axial view of the 2 random tiles that are shown in **a** and **b**. **f,** Higher magnification view of the randomly selected regions of **d** and **e**, marked by the corresponding colored square boxes. Scale bars, 120 µm (**a,b,d,e**); 25 µm (**c,f**).

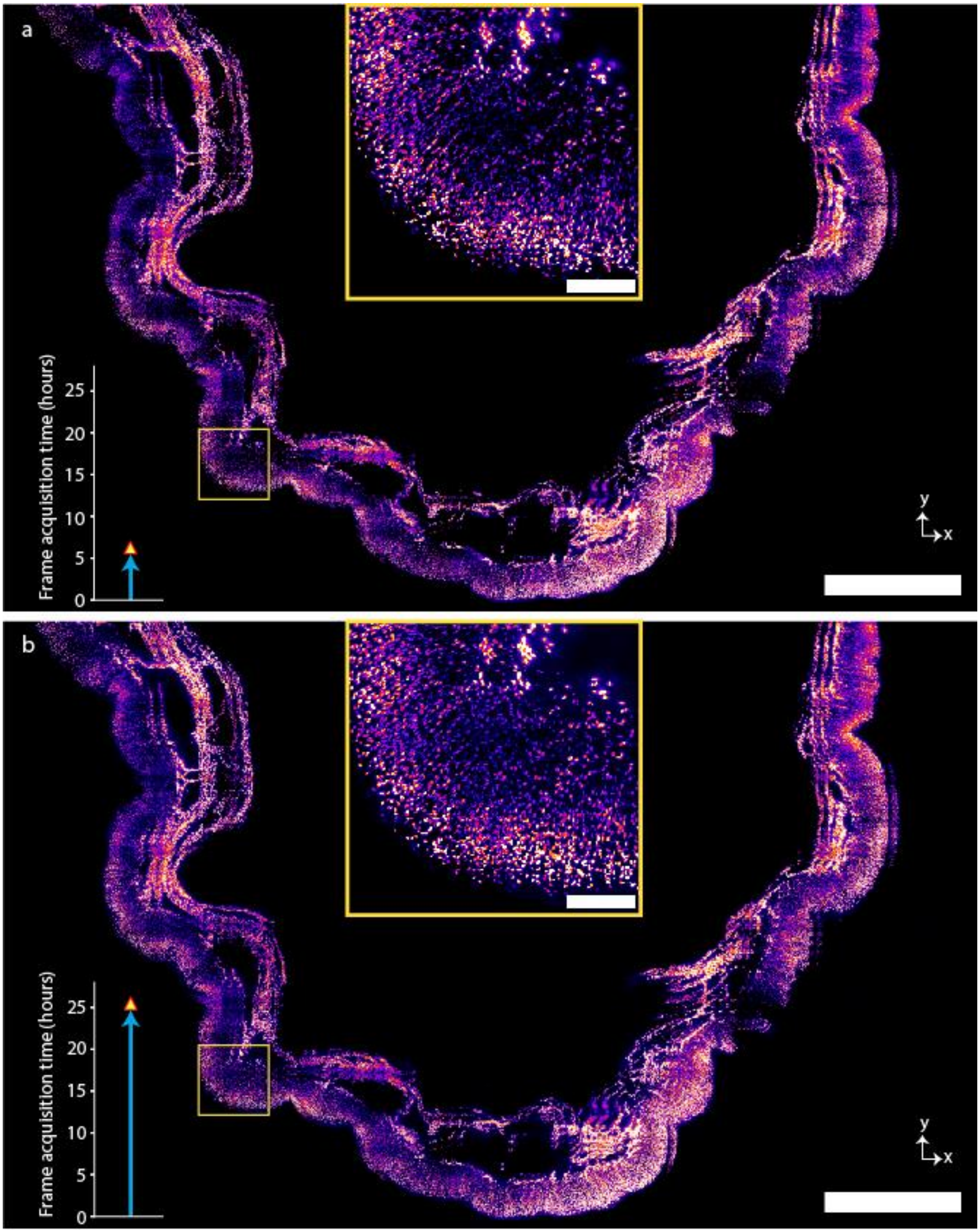

Supplementary Figure 17 | **Volumetric imaging of mouse stomach. a-b,** MIP of 100 slices of the whole stitched stomach, acquired by SIFT at 40 fps (**a**) and by traditional ASLM at 10 fps (**b**). Total frame acquisition time is reflected by the bar plot of the respective images. Scale bars, 1 mm (**a,b**), 120 μm (inset of **a,b)**.

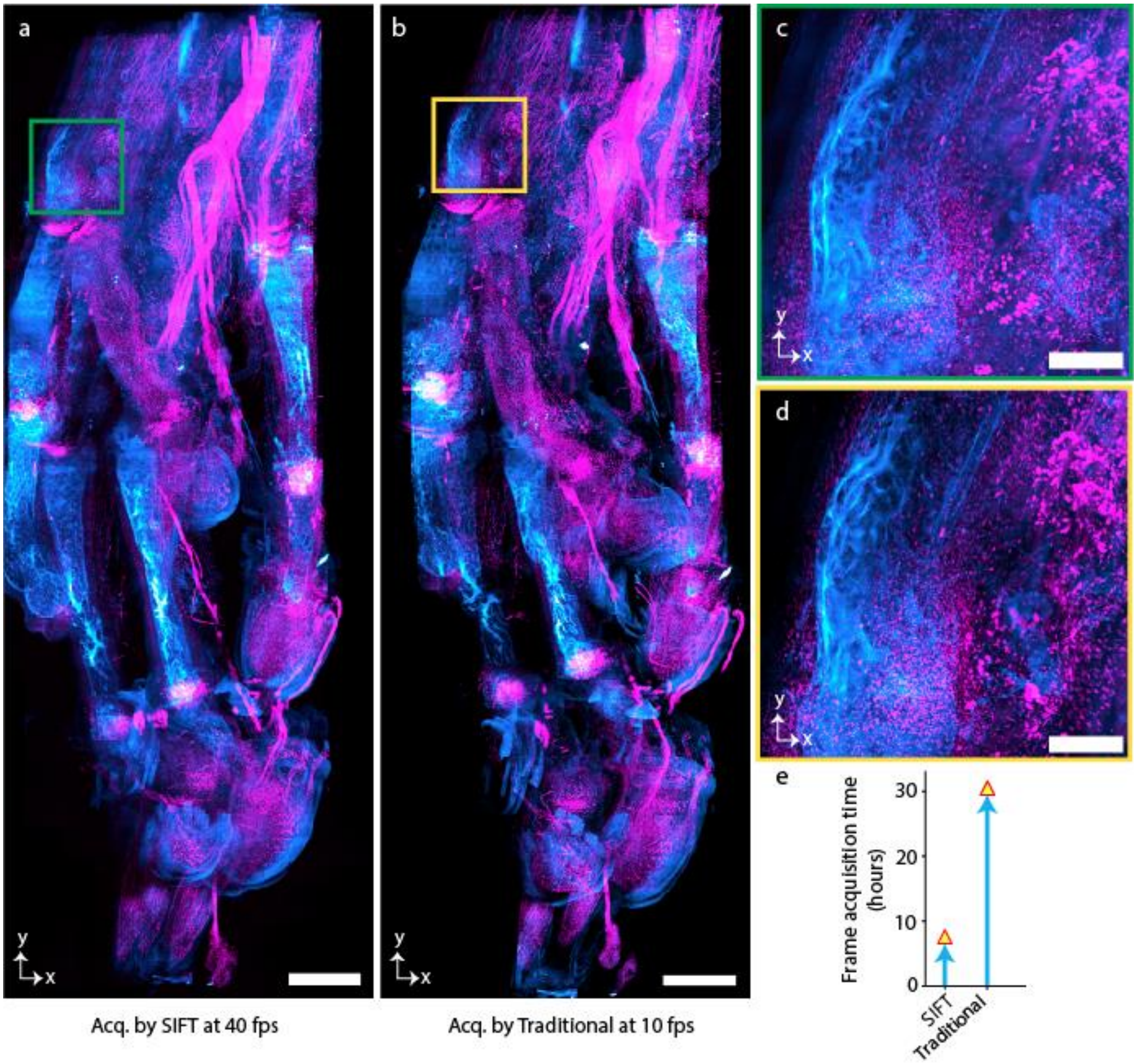

Supplementary Figure 18 | Volumetric imaging of mouse forepaw. a-b, MIP of Wnt1-cre Scarlett flow dual channel mouse forepaw, acquired by SIFT at 40 fps (**a**) and by traditional ASLM at 10 fps (**b**). **c-d,** Higher magnification view of the randomly selected region from **a** and **b** respectively. **e,** Comparison of total frame acquisition time to image the tissue using SIFT at 40 fps and traditional ASLM at 10 fps. Scale bars, 600 μm (**a, b**); 150 μm (**c,d**).

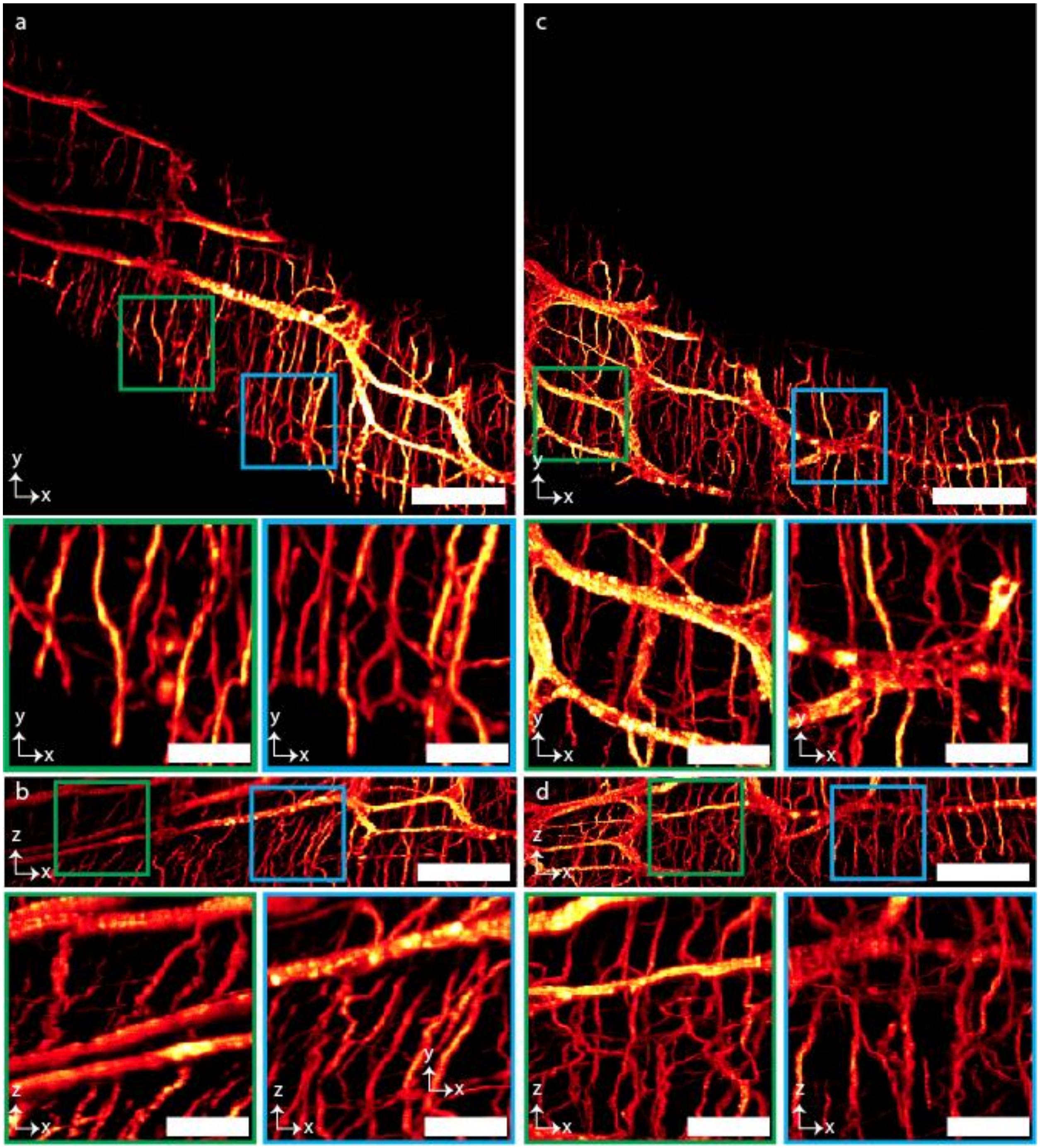

Supplementary Figure 19 | Volumetric imaging of mouse colon. a-b, Lateral (**a**) and axial (**b**) view of the MIP of a tile of the mouse colon and the enlarged view of the randomly selected regions shown in square boxes of **a** and **b. c-d,** Lateral (**c**) and axial (**d**) view of the MIP of another tile of the mouse colon and the enlarged view of the randomly selected regions shown in square boxes of **c** and **d**. Scale bars, 120 μm (**a-d**), enlarged view 40 μm.

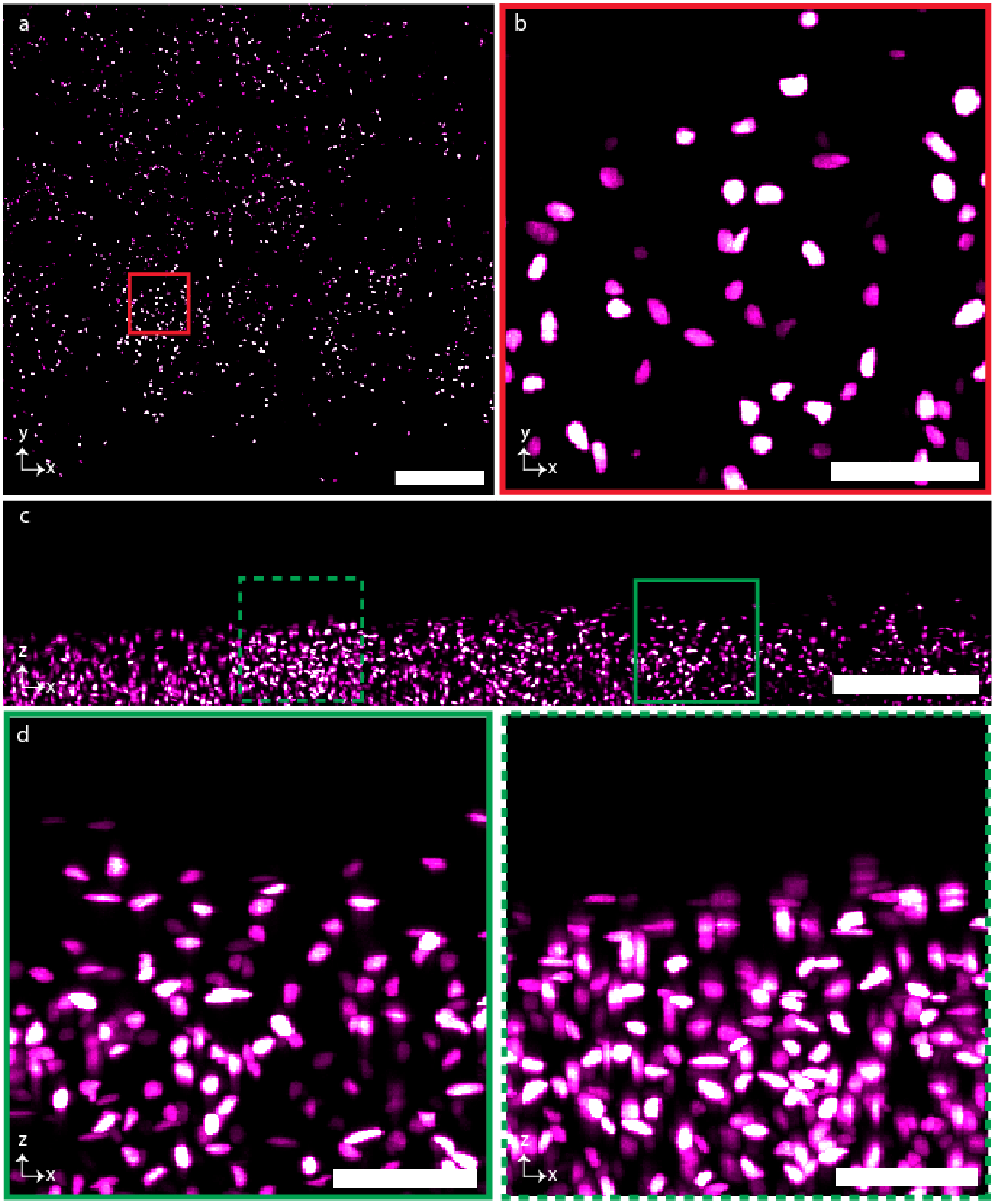

Supplementary Figure 20a | Representative tile of mouse stomach. a, MIP of the lateral view of a single tile among the 2,171 tiles of mouse stomach (shown stitched stomach in **Supplementary Figure 17** and **Supplementary Movie 1**). **b,** Higher magnification view of a randomly selected region from **a**. **c,** MIP of the axial view of the same tile. **d,** Higher magnification view of the selected regions of **c**. Scale bars, 120 μm (**a**); 45 μm (**b,d**); 100 μm (**c**).

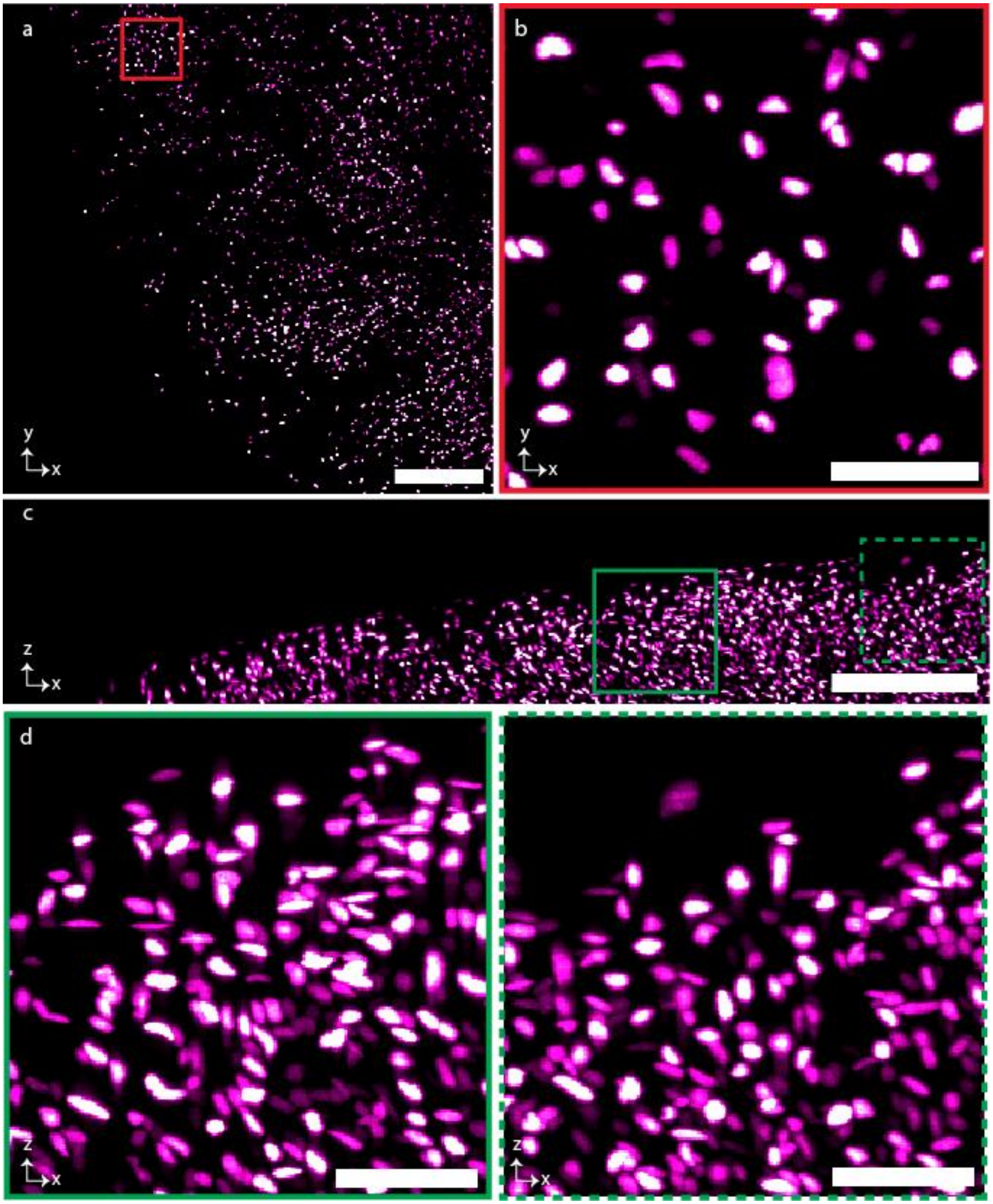

Supplementary Figure 20b | Representative 2nd tile of mouse stomach. a, MIP of the lateral view of a single tile among the 2,171 tiles of mouse stomach (shown stitched stomach in **Supplementary Figure 17** and **Supplementary Movie 1**). **b,** Higher magnification view of a randomly selected region from **a**. **c,** MIP of the axial view of the same tile. **d,** Higher magnification view of the selected regions of **c**. Scale bars, 120 μm (**a**); 45 μm (**b,d**)**;** 100 μm (**c**).

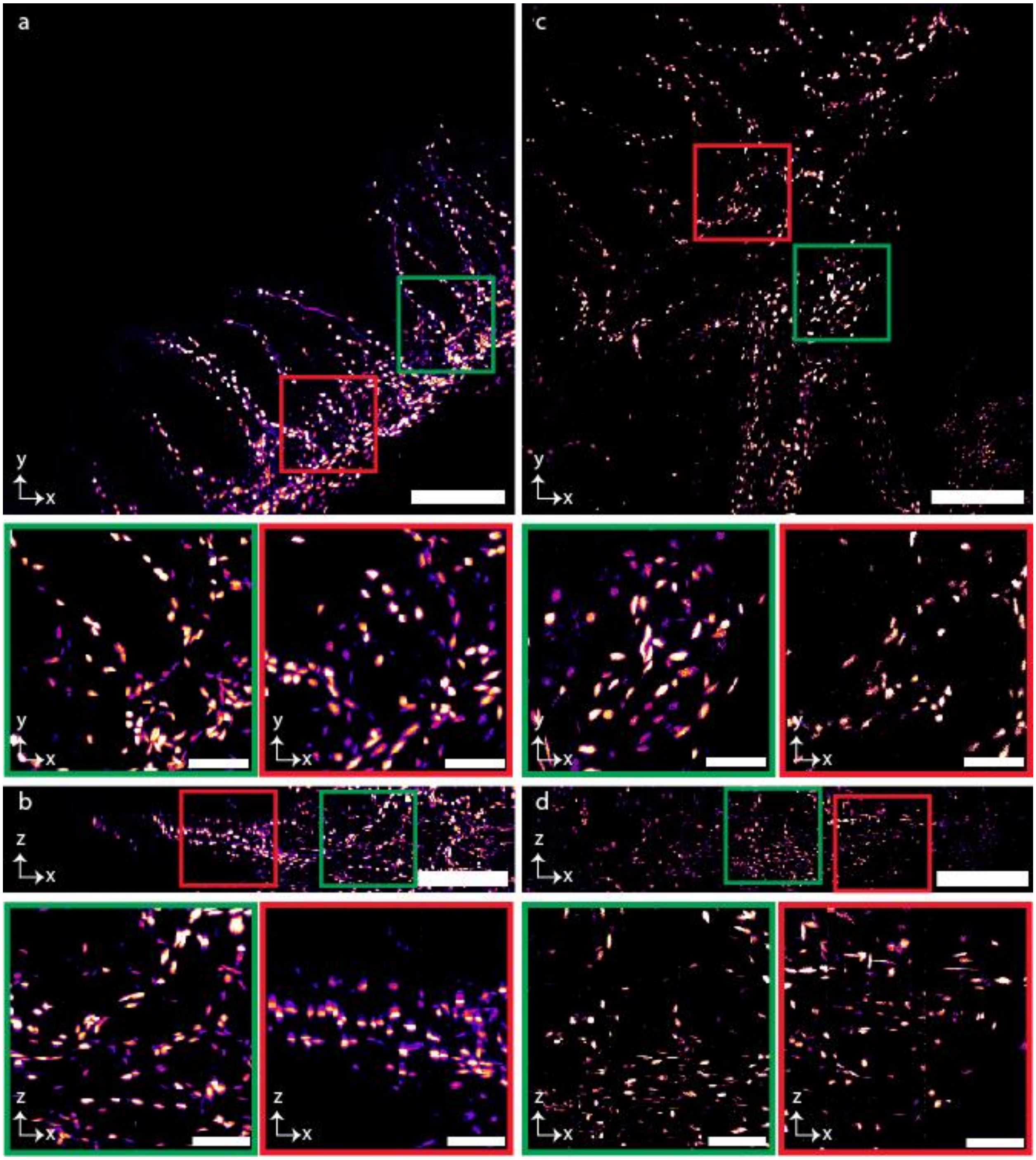

Supplementary Figure 21 | Image of different tiles of mouse gut. a-b, Lateral (**a**) and axial (**b**) view of the MIP of a tile of the mouse gut and the enlarged view of the randomly selected regions shown in square boxes of **a** and **b.** (shown stitched gut in **Supplementary Figure 14** and **Supplementary Movie 6**). **c-d,** Lateral (**c**) and axial (**d**) view of the MIP of another tile of the mouse gut and the enlarged view of the randomly selected regions shown in square boxes of **c** and **d**. Scale bars, 120 μm (**a-d**), 25 μm (enlarged view of **a-d**).

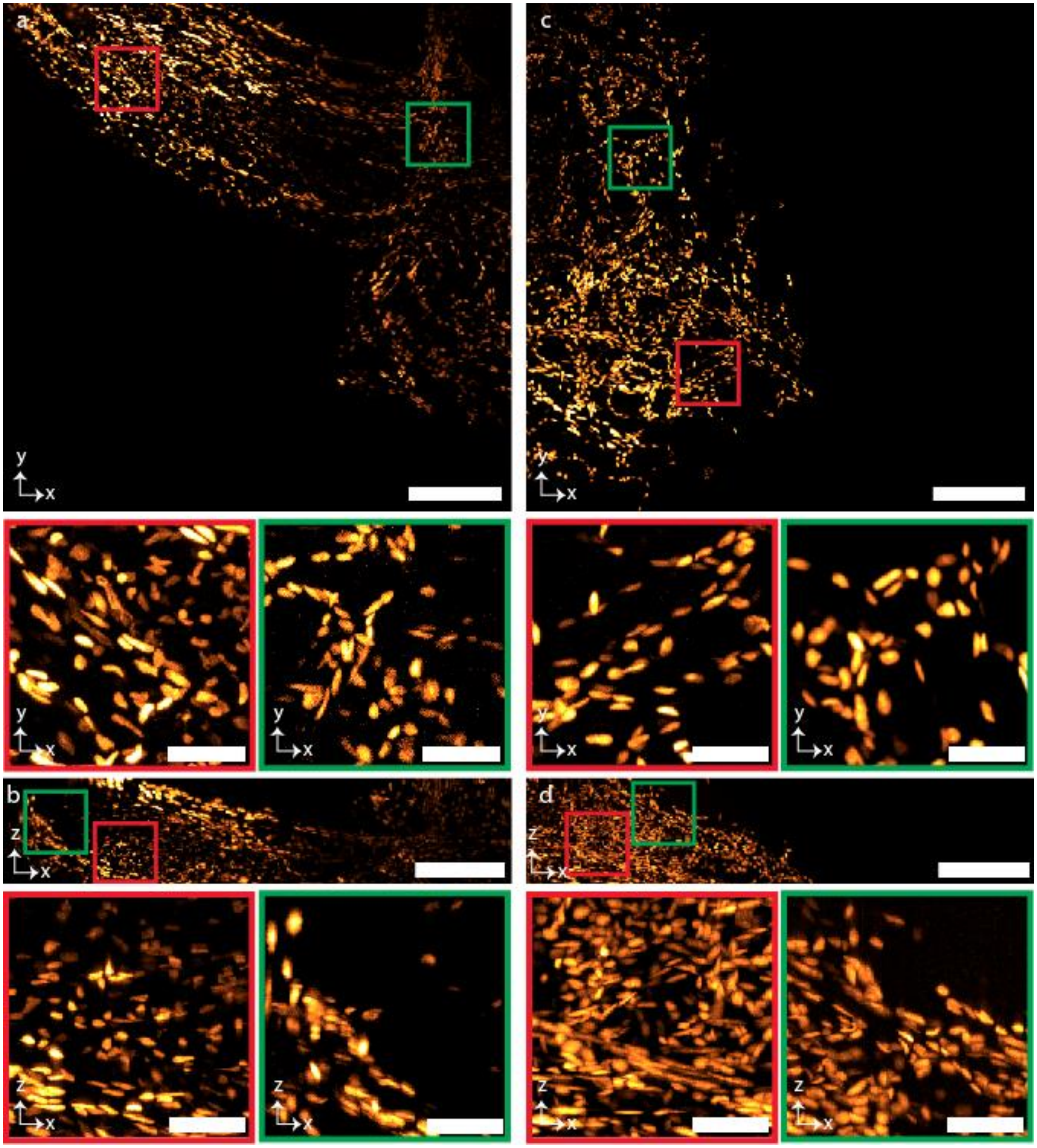

Supplementary Figure 22 | Image of different tiles of mouse hindpaw. a-b, Lateral (**a**) and axial (**b**) view of the MIP of a tile of the mouse forepaw and the enlarged view of the randomly selected regions shown in square boxes of **a** and **b. c-d,** Lateral (**c**) and axial (**d**) view of the MIP of another tile of the mouse forepaw and the enlarged view of the randomly selected regions shown in square boxes of **c** and **d**. Scale bars, 120 μm (**a-d**), 25 μm (enlarged view of **a-d**).

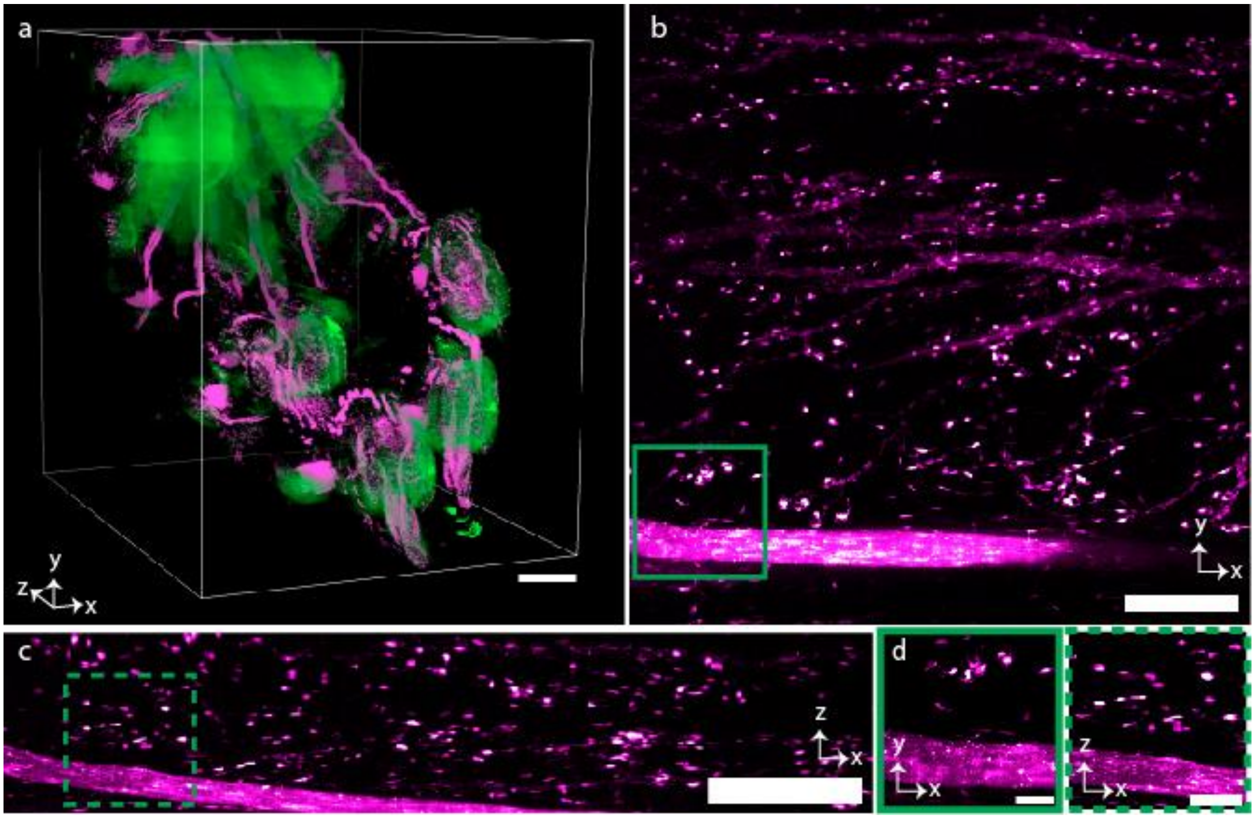

Supplementary Figure 23 | Volumetric imaging of mouse forepaw. a, 3D rendered view of dual channel mouse forepaw (**Fig. 3**) cleared by PEGASOS protocol and imaged by SIFT at 40 fps. The image volume is 4.2 x 3.3 x 5.5 mm^3. **b-c,** MIP of the lateral (**b**) and axial (**c**) view of one image tile. **d,** Enlarged view of the selected regions from **b** and **c**, marked by green square boxes. Scale bars, 500 μm (**a**); 120 μm (**b,c**); 30 μm (**d**).

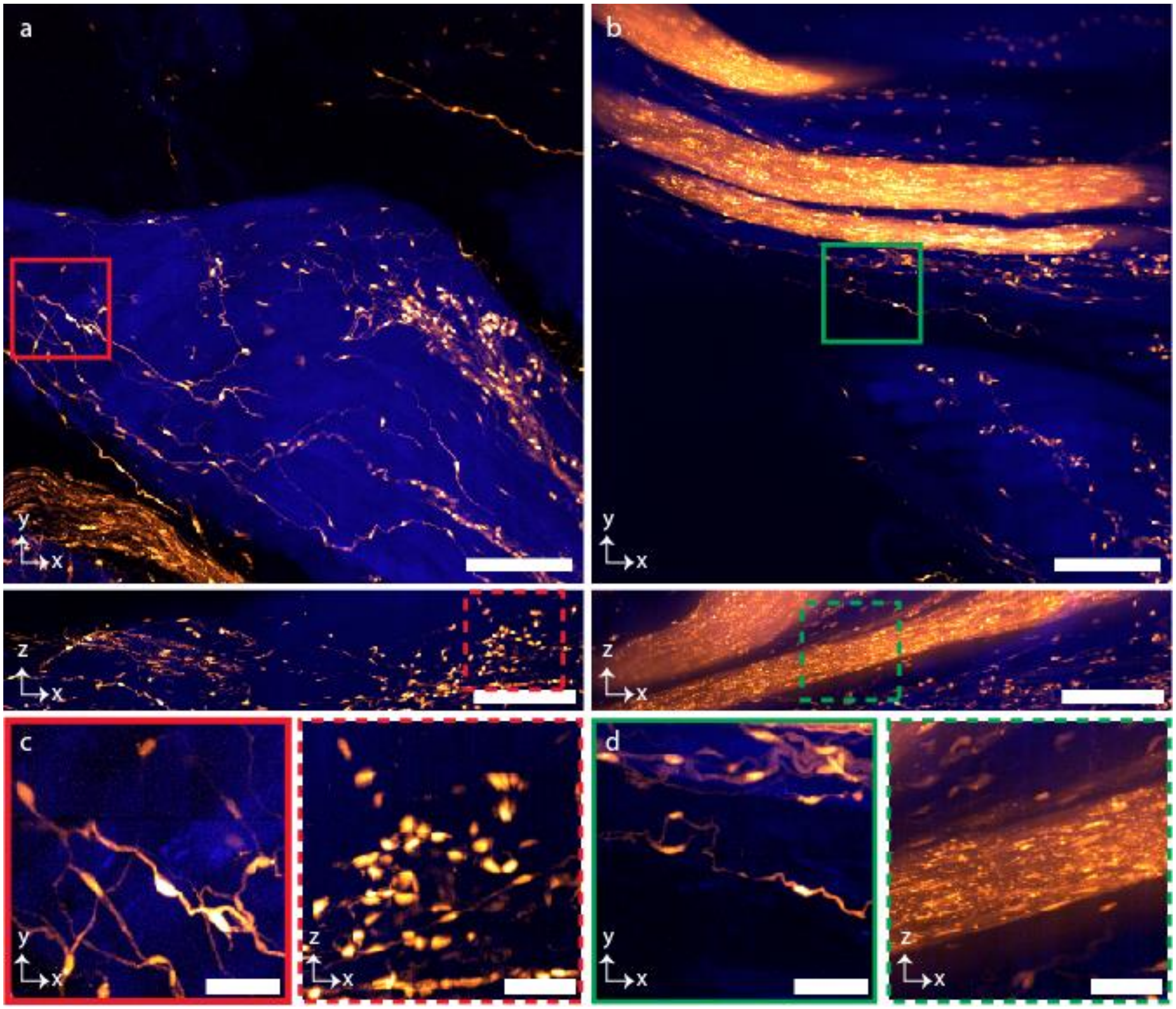

Supplementary Figure 24 | Image of different tiles of mouse forepaw. a-b, Lateral and axial view of the MIP of a tile of the mouse forepaw for two random tiles (whole stitched dual channel forepaw is shown in **Fig. 3**). **c-d,** Higher magnification view of randomly selected regions from **a** (**c**) and **b** (**d**). Scale bars, 120 μm (**a-b**), 30 μm (**c-d**).

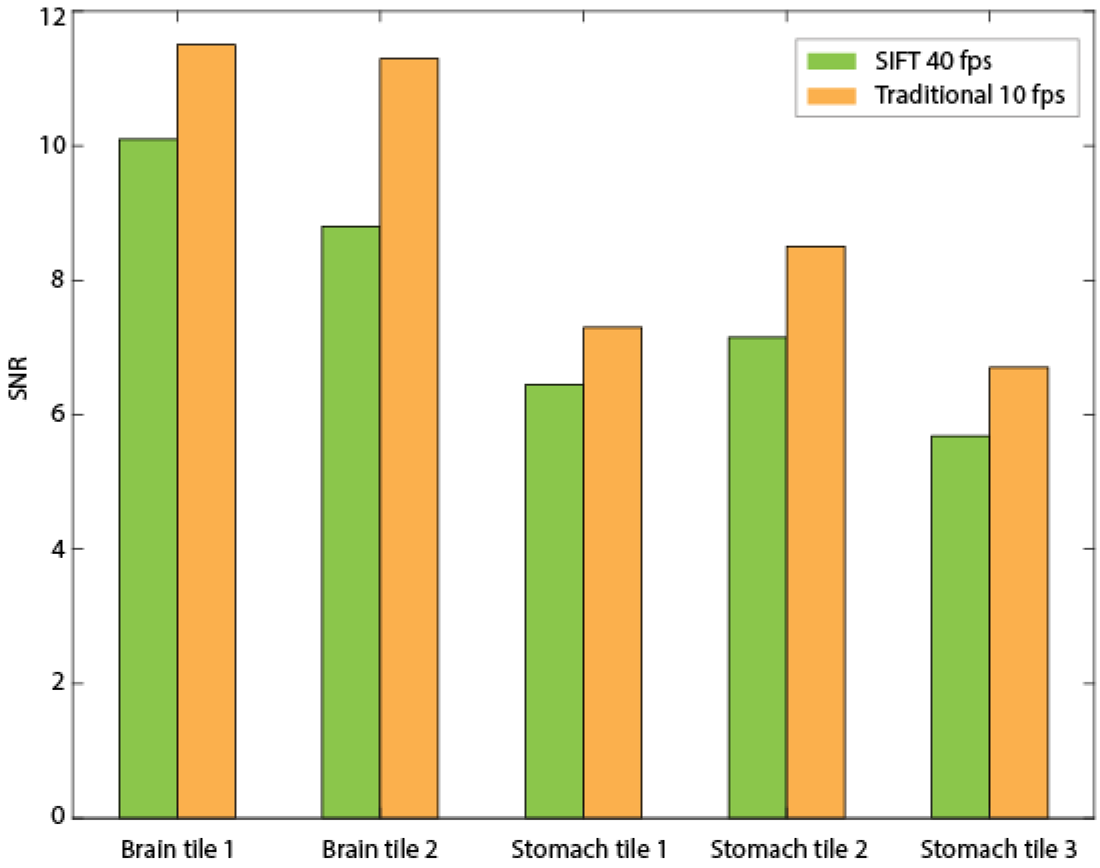

Supplementary Figure 25 | Comparison of SNR. Statistical data for signal to noise ratio (SNR), computed for randomly selected five tiles taken from two different specimens acquired by SIFT at 40 fps and traditional ASLM at 10 fps.

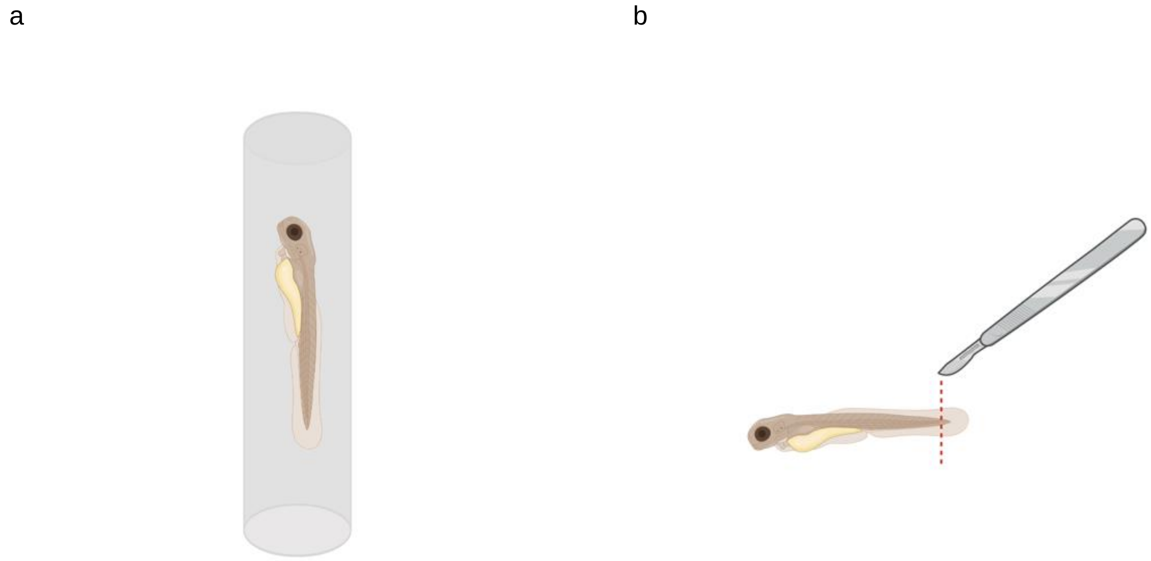

Supplementary Figure 26 | Imaging of live zebrafish. a, A live zebrafish larvae, anesthetized, embedded in agarose and then mounted in a FEP tube. **b,** Methods for zebrafish tail cut. The larvae were anesthetized in Tricaine (160 mg/L) and tails were cut with a sharp scalpel. Fish were then placed in low melt agarose at 42˚C before being sucked into a capillary tube made of FEP (ZEUS Virtual item: 0000183678).

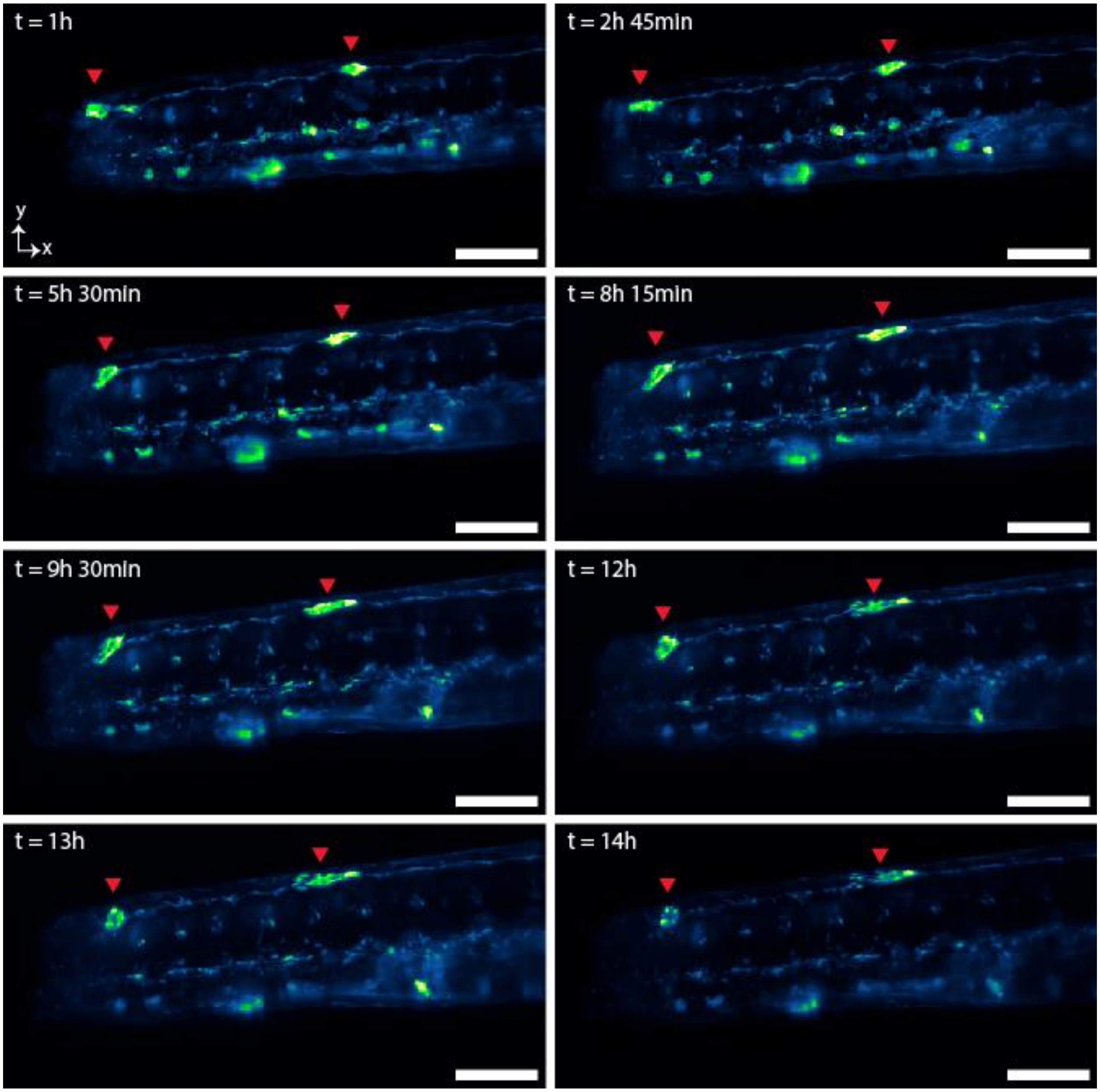

Supplementary Figure 27 | Additional z-depth of Zebrafish tail following injury. As seen in Fig. 4, ROI as marked by the red box shown in **Fig. 4a**, and images are of the same animal as seen in Fig. **4b** (different z-depth). Red arrows indicate regions of egfp expression with a migratory behavior or change in morphology. Scale bar, 100 μm.

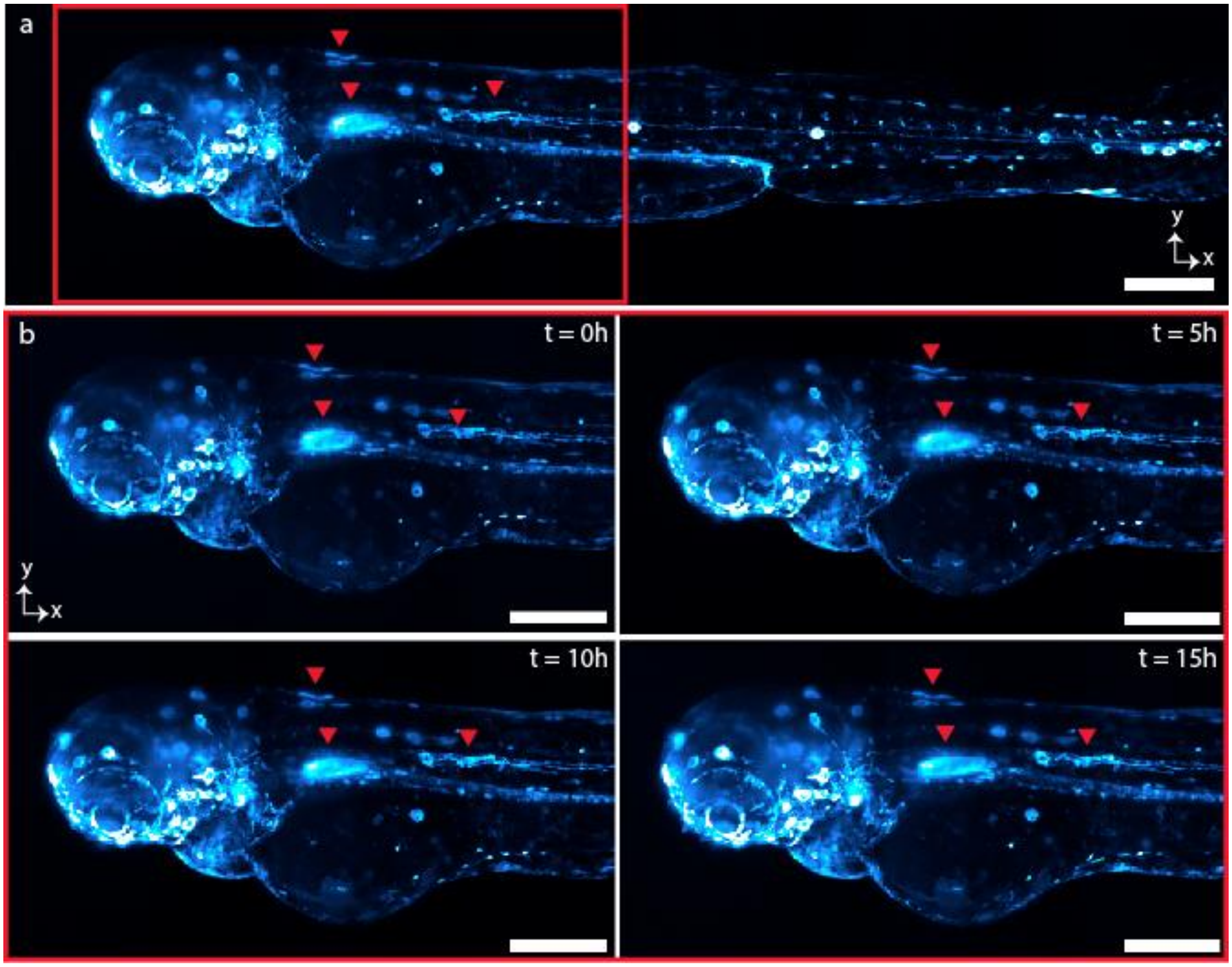

Supplementary Figure 28 | Time lapse of nfkb:egfp zebrafish development over 15 hours. a, MIP of the 10th time-point image of whole zebrafish. b, As previously reported, nfkb expression in the stomach increases during development[1] and egfp+ cells migrated over the course of the time lapse. In **Supplementary Movie 4**, egfp+ cells (likely microglia) are observed migrating from the yolk sac towards the head, consistent with previous observations about the origins of larval microglia in zebrafish[2]. Scale bars, 200 mm (a); 100 mm (b).

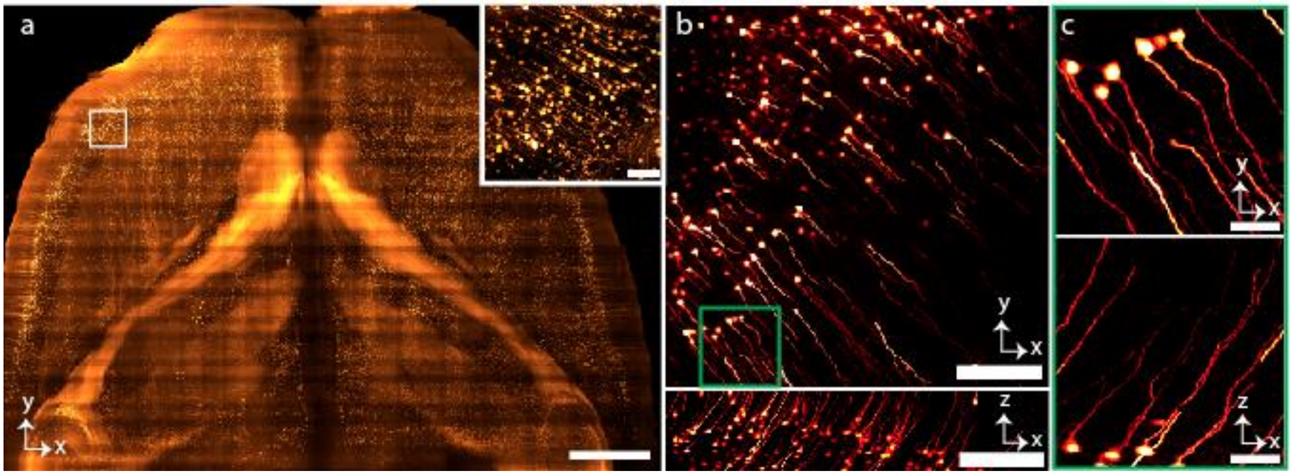

Supplementary Figure 29 | Imaging of mouse brain at RI ~1.56. a, MIP of the part of Thy-1 GFP neuronal mouse brain section cleared by PEGASOS protocol. The inset shows higher magnification view of the selected region. **b,** Lateral and axial views of a single tile showing the neuronal dendrites of the mouse brain. **c,** MIP of the higher magnification view in lateral and axial dimension corresponding to the region of the stack shown by the square green box (**b**). Scale bars, 1 mm (**a**), 80 µm (inset of **a**), 150 µm (**b**), 30 µm (**c**).

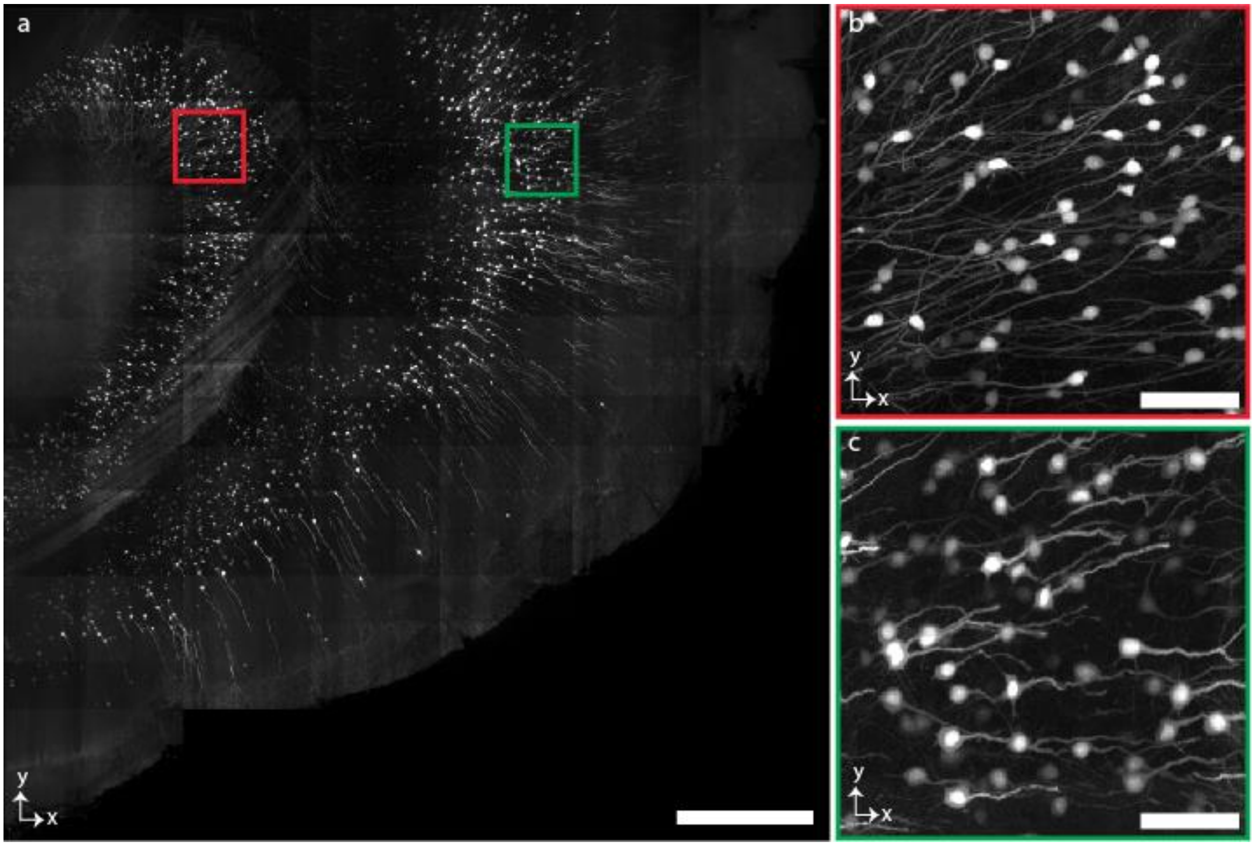

Supplementary Figure 30 | Image of Thy-1 GFP mouse brain. a, MIP of a part of Thy-1 GFP neuronal mouse brain. **c-b,** Higher magnification view of randomly selected regions corresponding to the red (**b**) and green (**c**) square boxes of **a**. Scale bars, 500 μm (**a**), 60 μm (**b-c**).

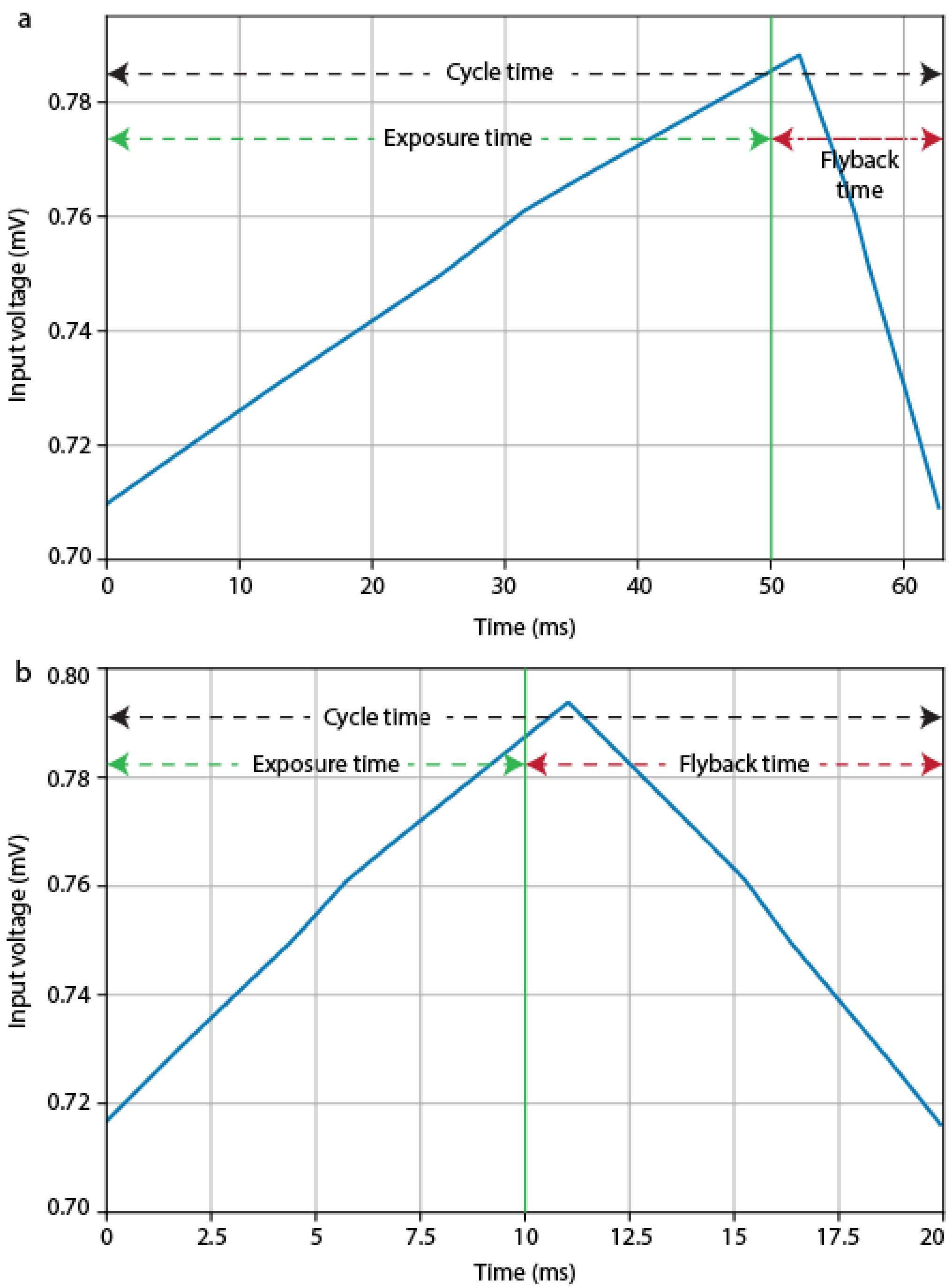

Supplementary Figure 31 | The sawtooth voltage signal for LFA. a-b, Synchronized TTL triggers for the camera and laser modulation at 50 ms (**a**) and 10 ms (**b**) of camera exposure time. LFA flyback time almost doesn’t changes with the decrement of camera exposure time. A signification flyback time causes increment of imaging time though the temporal resolution is equal to the camera exposure time.

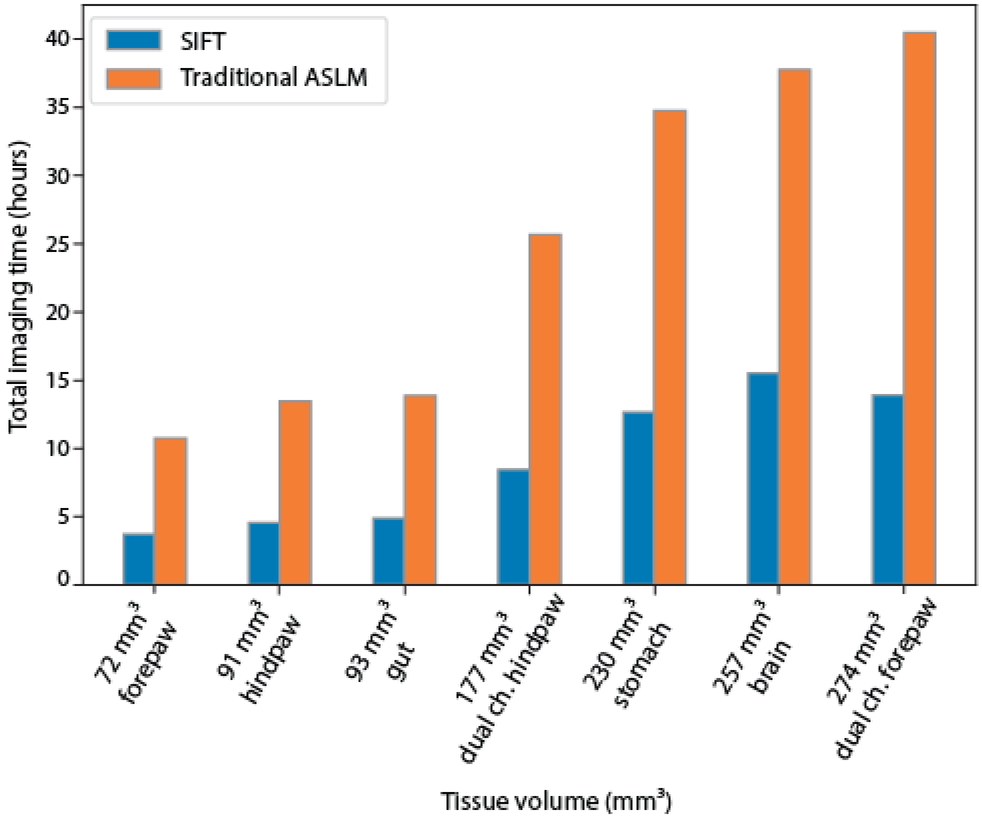

Supplementary Figure 32 | Total imaging time. Comparison of total imaging time for various tissue specimens having different shapes and volumes. This imaging time is for the equipment used in our experiment (**Supplementary Table 1**). Imaging time may vary depending on the response time of the filter wheel and the stage positional movement.

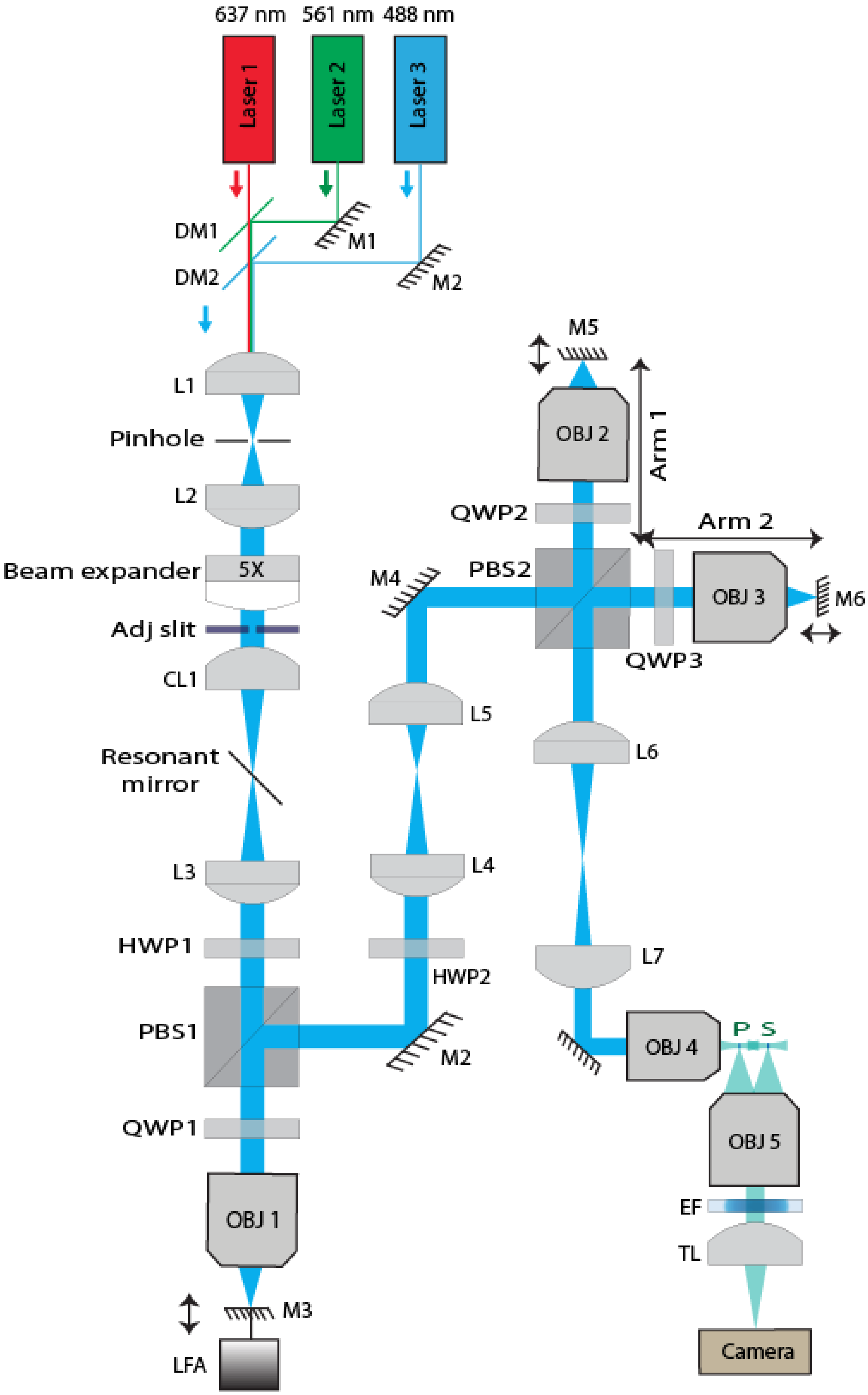

Supplementary Figure 33 | Schematic drawing of SIFT. Corresponding optical components are listed in Supplementary Table 1.

Supplementary Notes

Note 1 - LFA movement

Linear focus actuator (LFA) is a high-performance, small-footprint positioning actuator, specially designed for fast positioning with high precision during short to medium strokes. The voice coil motor is coupled with a high-precision feedback encoder. Low moving mass (50 grams), small step response time (< 3 millisecond) and precise positioning resolution (< 50 nanometers) make LFA useful to various applications such as medical imaging, optical engineering, electronic appliances, semiconductor industry etc.

A tiny mirror is attached in front of the LFA to translate the light-sheet. We found that the LFA movement is not linear with the input voltage over its total travel range[3]. As it is challenging to obtain a precise calibration of the LFA movement with respect to the input voltage, we selected a travel range where the LFA response is linear and covers the range required for imaging the full FOV. With LFA operated in a linear regime, the synchronization procedure becomes easier, where we only need to calibrate the minimum and the maximum voltages for the dual foci to cover the full FOV. And when imaging samples with different refractive indices, we only need to calibrate an offset voltage and keep the difference between the minimum and the maximum voltages the same.

Note 2 – Remote focusing technique

A perfect pupil matched remote focusing technique[4–6] is able to correct the aberrations introduced by higher-order polynomials. Though existing techniques such as acoustic lens[7], acoustic LS[8], multi-tiling imaging[9,10], electrotuneable lens (ETL)[11–14] reported to scan LS in the direction of propagation, those techniques introduce additional aberrations.

To achieve perfect imaging (free of aberrations), the remote focusing technique demands matching the pupil of the objectives through a 4f imaging system in order to reduce the spherical aberrations. To match the pupils of the two objectives, the magnification (M) of the 4f system must satisfy:

$$M = \frac{n_2 F_2 M_1}{n_1 F_1 M_2} \tag{1}$$

where, $M_{1,2}$, $F_{1,2}$ and $n_{1,2}$ represent the magnifications, the focal lengths of the designed tube lenses and the refractive indices of the immersion media for the two objectives. Therefore, a careful selection of the lenses for the 4f system is critical to minimize aberrations. An automatic lens selection software is available for designing the 4f system in remote focusing[15]. Besides reducing aberrations, remote focusing also allows us to image faster. The LFA is capable of sweeping the light-sheet over the entire camera chip (2048 x 2048 pixels) at a speed of 40 frames per second (fps).

Note 3 - Building SIFT system

The most promising fact of SIFT is its simple design. Although we add two additional RF objectives this makes optimization and troubleshooting easier. We expect the system can be built in two months by an experienced optical engineer. Considering a large tissue, several terabytes of the storage facility and data reading speed are required. The major equipment and parts are listed in **Supplementary Table 1**.

After initial construction, the system is first fine-tuned by generating two 2D focusing beams after the illumination objective and imaging the fluorescein dilution. The two foci are axially swept back-and-forth by LFA. A tight synchronization of the two foci with the camera rolling shutter was achieved by carefully adjusting the range and starting point of the LFA movement along with the flyback time and the cycle time for a specific camera exposure time (**Fig. 2c-2d** and **Supplementary Figure 31**). A tight synchronization results in a sharp, uniform line over the entire FOV (**Fig. 1**). After synchronization adjustment, a cylindrical lens is added to the illumination path, which generates the light-sheet. For the following alignment we used fluorescent beads embedded in agarose. The rotation and translation of the cylindrical lens were adjusted to achieve uniform bead brightness across the full FOV. The distance between the camera and the detection objective was adjusted to minimize the spherical aberration. A z stack of fluorescent beads was imaged and subsequently, full width half maximums (FWHMs) of the bead images were measured in both the lateral and axial dimensions.

Supplementary Tables

Supplementary Table 1 | Equipment list. Detail list of materials used to construct SIFT.

Sl. No.	Description	Item abbreviation	Part number	Part number company	Qty	Remarks
1	4 channel combo OBIS laser	Laser 0	LX 405-100C	Coherent	1	
		Laser 3	LX 488-50C			
		Laser 2	LX 561-50			
		Laser 1	LX 637-140C			
2	427 nm Dichroic beam splitter	DM3	LM01-427-25	Semrock	1	
3	503 nm Dichroic beam splitter	DM2	LM01-503-25	Semrock	1	
4	613 nm Dichroic beam splitter	DM1	LM01-613-25	Semrock	1	
5	f = 50 mm, Ø1" achromatic doublet	L1	AC254-75-A	ThorLabs	1	
6	30 µm pinhole	Pinhole	P30D	ThorLabs	1	
7	f = 75 mm, Ø1" achromatic doublet	L2	AC254-50-A	ThorLabs	1	
8	5X Galilean beam expander	Beam expander	GEB05-A	ThorLabs	1	
9	Adjustable mechanical slit	Adj slit	VA100	ThorLabs	1	
10	f = 50 mm, Ø1" cylindrical achromat	CL1	ACY254-50-A	ThorLabs	1	
11	High precision rotation stage	Rot stage	PR01	ThorLabs	1	
12	Resonant mirror galvanometer	Resonant mirror	CRS 4 kHz	Cambridge Technology	1	
13	12V DC power supply	12V DC	A12MT400	Acopian	1	
14	f = 200 mm, Ø2" achromatic doublet	L3	AC508-200-A	ThorLabs	1	
15	Half wave plate	HWP	AHWP3	ThorLabs	2	
16	Polarizing beam splitter	PBS1	10FC16PB.7	Newport	2	
17	Ø1" protected silver mirror	M	PF10-03-P01	ThorLabs	5	
18	1" x 1" protected silver mirror	M	PFSQ10-03-P01	ThorLabs	3	
19	2" x 2" protected silver mirror	M	PFSQ20-03-P01	ThorLabs	2	
20	Quarter wave plate	QWP	AQWP3	ThorLabs	3	
21	Microscope objective (x4, NA 0.28)	OBJ1	XL Fluor x4	Olympus Life Sciences	1	
22	Linear focus actuator (LFA)	LFA	LFA-2010	Equipment Solutions	1	
23	N-BK7 glass piece	GP	37-005	Edmund optics	1	
24	Self-Contained XYZ 25 mm translation stage	3D Tran stage	LX30	ThorLabs	2	
25	f = 200 mm, Ø2" achromatic doublet	L4, L6	ACT508-200-A-ML	ThorLabs	2	
26	f = 75 mm, Ø2" achromatic doublet	L5	AC508-075-A-ML	ThorLabs	1	

Sl. No.	Description	Item abbreviation	Part number	Part number company	Qty	Remarks
27	Microscope objective (x10, NA 0.30)	OBJ2, OBJ3		Olympus UMP PlanFl	2	
28	50 mm travel linear translation stage	Lin Tran stage1	XR50P	ThorLabs	2	
29	1/4" travel single axis translation stage	Lin Tran stage2	MS1S	ThorLabs	2	
30	2" travel single axis translation stage	Lin Tran stage3	LT1	ThorLabs	1	
31	f = 150 mm, Ø2" achromatic doublet	L7	AC508-150-A-ML	ThorLabs	1	
32	Cleared tissue objective (16.7x/0.4, RI 1.45)	OBJ4	Special Optics 54-10-12	Applied scientific instrumentation	2	
33	f = 200 mm, Ø2" tube lens	OBJ5	ITL200-A	ThorLabs	1	
34	Emission filter	EF1	FF01-525/30-25	Semrock	1	
35	Emission filter	EF2	FF01-605/15-25	Semrock	1	
36	Emission filter	EF3	BPL01-647R-25	Semrock	1	
37	3D motorized stage	Mot stage	Model: MP-285A, PCIe 80 7852R	National Instruments	1	
38	4 position filter wheel	Wheel	LAMBDA 10-B	Sutter Instrument	1	
39	Digital sCMOS camera	Camera	Orca Flash 4.0, V2<br>model: C13440-20CU	Hamamatsu Corporation	1	

Qty. quantity

Supplementary Table 2 | Tissue imaging. Detail of tissues, imaged using the pipeline.

Sl. no.	Tissues	Volume imaged (mm^3)	No. of tiles	Storage requirement (TB)	Clearing protocol	RI of clearing media
1	Mouse forepaw	3.7 x 3.5 x 2.5	685	2.22	Pegasos	1.56
2	Mouse hind paw	3.8 x 4.2 x 2.8	778	2.54	Pegasos	1.56
3	Mouse gut	5.5 x 4.6 x 2.8	878	2.84	Pegasos	1.56
4	Dual channel mouse hind paw	4.2 x 3.3 x 5.5	1,672	5.40	Pegasos	1.56
5	Mouse stomach	4.4 x 7.1 x 3.2	2,171	7.03	Pegasos	1.56
7	Nuclear stained Mouse brain	10.4 x 6.1 x 3.2	2,426	7.86	Pegasos	1.56
8	Dual channel mouse forepaw	8.5 x 3.1 x 4.25	2,588	8.38	Pegasos	1.56
9	Thy-1 GFP neuronal mouse brain	11.2 x 8.4 x 6.43	2,366	7.67	Pegasos	1.56
10	Thy1-YFP-H mouse brain	17 x 12 x 3.43	4,111	11.80	CUBIC-L/R	1.52
11	Mouse colon	3.7 x 3.7 x 0.23	36	0.123	Sca*l*eCUBIC	1.48
12	Zebrafish	2.8 x 0.7 x 0.62	42	0.139	Uncleared	1.33

Supplementary Table 3 | Multi-immersion imaging. Detail imaging parameters for multiple tissue immersion media.

Sl. no.	Refractive Index	Pixel size (μm)	Magnification (x)	FOV (μm^2)	Remarks
1	Water (~ 1.33)	0.425	15.28x	870 x 870	
2	CLARITY (~ 1.44)	0.38	17.10x	775 x 775	
3	CUBIC-R (~ 1.52)	0.37	17.70x	750 x 750	
4	Tocris (~ 1.53)	0.365	17.80x	745 x 745	
5	PEGASOS (~ 1.56)	0.36	18x	740 x 740	

Supplementary Table 4 | Cost of tissue imaging. Comparison between SIFT and the traditional ASLM in terms of the cost of imaging.

Sl. no.	Tissues	Imaging cost (appr.)[a,b]	
		SIFT	Traditional ASLM
1	Mouse forepaw	$ 131	$ 376
2	Mouse hind paw	$ 160	$ 471
3	Mouse gut	$ 171	$ 485
4	Dual channel mouse hind paw	$ 295	$ 899
5	Mouse stomach	$ 443	$ 1,217
7	Nuclear stained mouse brain	$ 542	$ 1,322
8	Dual channel mouse forepaw	$ 487	$ 1,417
9	Mouse colon	$ 17	$ 50
10	Zebrafish	$ 8	$ 23

Appr. Approximated

[a]Included approximated high resolution imaging cost (not included the low resolution and structural evaluation time)

[b]Approximately $35 per hour fluorescence imaging[16–18]

Supplementary references

1. Kanther, M. *et al.* Microbial Colonization Induces Dynamic Temporal and Spatial Patterns of NF-κB Activation in the Zebrafish Digestive Tract. *Gastroenterology* **141**, 197–207 (2011).
2. Xu, J. *et al.* Temporal-Spatial Resolution Fate Mapping Reveals Distinct Origins for Embryonic and Adult Microglia in Zebrafish. *Developmental Cell* **34**, 632–641 (2015).
3. Dibaji, H., Prince, M. N. H., Yi, Y., Zhao, H. & Chakraborty, T. Axial scanning of dual focus to improve light sheet microscopy. *Biomed. Opt. Express, BOE* **13**, 4990–5003 (2022).
4. Botcherby, E. J., Juskaitis, R., Booth, M. J. & Wilson, T. Aberration-free optical refocusing in high numerical aperture microscopy. *Opt. Lett., OL* **32**, 2007–2009 (2007).
5. Botcherby, E. J. *et al.* Aberration-free three-dimensional multiphoton imaging of neuronal activity at kHz rates. *Proceedings of the National Academy of Sciences* **109**, 2919–2924 (2012).
6. Botcherby, E. J., Juškaitis, R., Booth, M. J. & Wilson, T. An optical technique for remote focusing in microscopy. *Optics Communications* **281**, 880–887 (2008).
7. Dean, K. M. & Fiolka, R. Uniform and scalable light-sheets generated by extended focusing. *Opt. Express, OE* **22**, 26141–26152 (2014).
8. Wunderl, S. *et al.* Acoustic light-sheet microscopy. 2021.08.20.457051 Preprint at https://doi.org/10.1101/2021.08.20.457051 (2021).
9. Fu, Q., Martin, B. L., Matus, D. Q. & Gao, L. Imaging multicellular specimens with real-time optimized tiling light-sheet selective plane illumination microscopy. *Nat Commun* **7**, 11088 (2016).
10. Chen, Y. *et al.* A Versatile Tiling Light Sheet Microscope for Imaging of Cleared Tissues. *Cell Reports* **33**, 108349 (2020).
11. Fahrbach, F. O., Voigt, F. F., Schmid, B., Helmchen, F. & Huisken, J. Rapid 3D light-sheet microscopy with a tunable lens. *Opt. Express, OE* **21**, 21010–21026 (2013).
12. Hedde, P. N. & Gratton, E. Selective plane illumination microscopy with a light sheet of uniform thickness formed by an electrically tunable lens. *Microscopy Research and Technique* **81**, 924–928 (2018).
13. Voigt, F. F. *et al.* The mesoSPIM initiative: open-source light-sheet microscopes for imaging cleared tissue. *Nat Methods* **16**, 1105–1108 (2019).
14. Liu, Y., Rollins, A. M. & Jenkins, M. W. CompassLSM: axially swept light-sheet microscopy made simple. *Biomed. Opt. Express* **12**, 6571 (2021).
15. Hong, W. & Dunsby, C. Automatic tube lens design from stock optics for microscope remote-refocusing systems. *Opt. Express* **30**, 4274 (2022).
16. Cost Estimates & Fees | Microscopy. *U-M Biomedical Research Core Facilities* https://brcf.medicine.umich.edu/cores/microscopy/cost-estimates-fees/.
17. Recharge Rates. *Center for Cellular Imaging* https://wucci.wustl.edu/user-info/rates/ (2019).
18. User Fees | W.M. Keck Center for Cellular Imaging, U.Va. https://kcci.virginia.edu/facility/user-fees.
