# Supplementary material for: Signal improved ultra-fast light-sheet microscope for large tissue imaging
Source: Commun Eng. 2024 Apr 2;3:59. doi: 10.1038/s44172-024-00205-4 (PMC10987599; doi:10.1038/s44172-024-00205-4)
Supplement: Supplementary file 3 — Description of Additional Supplementary Files [file 44172_2024_205_MOESM3_ESM.pdf]

Description of Additional Supplementary Files

File name: Supplementary Video 1

Description: Volumetric rendering of PEGASOS cleared mouse stomach

File name: Supplementary Video 2

Description: Inflammatory cell movement in Zebrafish following tail injury at position_1

File name: Supplementary Video 3

Description: Inflammatory cell movement in Zebrafish following tail injury at position_2

File name: Supplementary Video 4

Description: Development of Zebrafish over time

File name: Supplementary Video 5

Description: Volumetric rendering of the proximal segments of mouse colon

File name: Supplementary Video 6

Description: Volumetric rendering of PEGASOS cleared mouse gut
