# Supplementary material for: Signal improved ultra-fast light-sheet microscope for large tissue imaging
Source: Commun Eng. 2024 Apr 2;3:59. doi: 10.1038/s44172-024-00205-4 (PMC10987599; doi:10.1038/s44172-024-00205-4)
Supplement: Supplementary file 10 — Reporting Summary [file 44172_2024_205_MOESM10_ESM.pdf]

nature portfolio

Corresponding author(s): Tonmoy Chakraborty

Last updated by author(s): Mar 14, 2024

Reporting Summary

Nature Portfolio wishes to improve the reproducibility of the work that we publish. This form provides structure for consistency and transparency in reporting. For further information on Nature Portfolio policies, see our Editorial Policies and the Editorial Policy Checklist.

Statistics

For all statistical analyses, confirm that the following items are present in the figure legend, table legend, main text, or Methods section.

n/a	Confirmed	
☐	☒	The exact sample size (*n*) for each experimental group/condition, given as a discrete number and unit of measurement
☐	☒	A statement on whether measurements were taken from distinct samples or whether the same sample was measured repeatedly
☐	☒	The statistical test(s) used AND whether they are one- or two-sided<br>*Only common tests should be described solely by name; describe more complex techniques in the Methods section.*
☒	☐	A description of all covariates tested
☒	☐	A description of any assumptions or corrections, such as tests of normality and adjustment for multiple comparisons
☐	☒	A full description of the statistical parameters including central tendency (e.g. means) or other basic estimates (e.g. regression coefficient) AND variation (e.g. standard deviation) or associated estimates of uncertainty (e.g. confidence intervals)
☒	☐	For null hypothesis testing, the test statistic (e.g. *F*, *t*, *r*) with confidence intervals, effect sizes, degrees of freedom and *P* value noted<br>*Give P values as exact values whenever suitable.*
☒	☐	For Bayesian analysis, information on the choice of priors and Markov chain Monte Carlo settings
☒	☐	For hierarchical and complex designs, identification of the appropriate level for tests and full reporting of outcomes
☒	☐	Estimates of effect sizes (e.g. Cohen's *d*, Pearson's *r*), indicating how they were calculated

Our web collection on statistics for biologists contains articles on many of the points above.

Software and code

Policy information about availability of computer code

Data collection	Labview control software that was developed by Coleman technologies
Data analysis	A custom-written intensity based (MATLAB) and deep learning based (Python) classification softwares are developed for analysis. The softwares along with the dependencies and the trained checkpoint are freely available in github page. Bigstitcher and stardist plugin of Fiji were used to stitch image volumes and segment nuclei respectively.

For manuscripts utilizing custom algorithms or software that are central to the research but not yet described in published literature, software must be made available to editors and reviewers. We strongly encourage code deposition in a community repository (e.g. GitHub). See the Nature Portfolio guidelines for submitting code & software for further information.

Data

Policy information about availability of data

All manuscripts must include a data availability statement. This statement should provide the following information, where applicable:

- Accession codes, unique identifiers, or web links for publicly available datasets
- A description of any restrictions on data availability
- For clinical datasets or third party data, please ensure that the statement adheres to our policy

The provided Github repository contains trained best checkpoint. The raw dataset used for this study is available upon request to the corresponding author.

Human research participants

Policy information about studies involving human research participants and Sex and Gender in Research.

Reporting on sex and gender	Sex and gender based analyses were not performed
Population characteristics	Research is independent to the population characteristics
Recruitment	N/A
Ethics oversight	The Janelia Farms Research Campus of the Howard Hughes Medical Institute has licensed a few critical components as well as a few procedures under the agreement of material transfer. Animal experiments for PEGASOS cleared specimen were approved by the Institutional Animal Care and Use Committee of the Chinese Institute for Brain Research. Experiments for CUBIC R cleared specimen were approved by Juntendo University (1569-2022279 and 1372-2022211), and National Institute for Physiological Sciences (22A044).

Note that full information on the approval of the study protocol must also be provided in the manuscript.

Field-specific reporting

Please select the one below that is the best fit for your research. If you are not sure, read the appropriate sections before making your selection.

☒ Life sciences ☐ Behavioural & social sciences ☐ Ecological, evolutionary & environmental sciences

For a reference copy of the document with all sections, see nature.com/documents/nr-reporting-summary-flat.pdf

Life sciences study design

All studies must disclose on these points even when the disclosure is negative.

Sample size	Microscope's spatial resolution was measured by imaging 500 nm fluorescence beads embedded in agarose gel (n=10). Various biological tissues such as dual channel mouse fore paw, dual channel mouse hind paw, mouse gut, mouse stomach and whole mouse brain cleared by different protocols, mouse colon were imaged using the same pipeline.
Data exclusions	No data were excluded intentionally.
Replication	Replication of the experiment was successful
Randomization	Not applicable
Blinding	Not applicable

Reporting for specific materials, systems and methods

We require information from authors about some types of materials, experimental systems and methods used in many studies. Here, indicate whether each material, system or method listed is relevant to your study. If you are not sure if a list item applies to your research, read the appropriate section before selecting a response.

Materials & experimental systems

n/a	Involved in the study	
☐	☒	Antibodies
☒	☐	Eukaryotic cell lines
☒	☐	Palaeontology and archaeology
☐	☒	Animals and other organisms
☒	☐	Clinical data
☒	☐	Dual use research of concern

Methods

n/a	Involved in the study	
☒	☐	ChIP-seq
☒	☐	Flow cytometry
☒	☐	MRI-based neuroimaging

Antibodies

Antibodies used	Mouse Cdh5-cre Ai14 H2BGFP transgenic line, R26-mScarlettflox

Antibodies used	C57BL/6J wildtype mouse: anti-Tubulin beta 3 pan-neuronal marker<br>Mouse Thy1-YFP-H
Validation	Experimetally validated

Animals and other research organisms

Policy information about studies involving animals; ARRIVE guidelines recommended for reporting animal research, and Sex and Gender in Research

Laboratory animals	Mice with genotypes including Thy1-EGFP-M (JAX# 007788), Ai14 (JAX# 007908), tTAflox (JAX# 008600), tetO-H2BGFP (JAX# 005104) and Wnt1-Cre2 (JAX# 022501), Tg(Cdh5-CreERT2) mice (Strain NO.T014691).<br>9-week-old female Thy1-YFP-H Tg mice (B6.Cg-Tg(Thy1-YFP)HJrs/J.
Wild animals	C57BL/6J wildtype mouse
Reporting on sex	N/A
Field-collected samples	N/A
Ethics oversight	Animal experiments for PEGASOS cleared specimen were approved by the Institutional Animal Care and Use Committee of the Chinese Institute for Brain Research. Experiments for CUBIC R cleared specimen were approved by Juntendo University (1569-2022279 and 1372-2022211), and National Institute for Physiological Sciences (22A044).

Note that full information on the approval of the study protocol must also be provided in the manuscript.
